# OpaR Controls a Network of Downstream Transcription Factors in *Vibrio parahaemolyticus* BB22OP

**DOI:** 10.1371/journal.pone.0121863

**Published:** 2015-04-22

**Authors:** Alison Kernell Burke, Leah T. C. Guthrie, Thero Modise, Guy Cormier, Roderick V. Jensen, Linda L. McCarter, Ann M. Stevens

**Affiliations:** 1 Department of Biological Sciences, Virginia Tech, Blacksburg, VA, United States of America; 2 Georgia Advanced Computing Resource Center, University of Georgia, Athens, GA, United States of America; 3 Department of Microbiology, University of Iowa, Iowa City, IA, United States of America

## Abstract

*Vibrio parahaemolyticus* is an emerging world-wide human pathogen that is associated with food-borne gastroenteritis when raw or undercooked seafood is consumed. Expression of virulence factors in this organism is modulated by the phenomenon known as quorum sensing, which permits differential gene regulation at low versus high cell density. The master regulator of quorum sensing in *V*. *parahaemolyticus *is OpaR. OpaR not only controls virulence factor gene expression, but also the colony and cellular morphology associated with growth on a surface and biofilm formation. Whole transcriptome Next Generation sequencing (RNA-Seq) was utilized to determine the OpaR regulon by comparing strains BB22OP (*opaR* ^+^, LM5312) and BB22TR (∆*opaR1*, LM5674). This work, using the published *V*. *parahaemolyticus* BB22OP genome sequence, confirms and expands upon a previous microarray analysis for these two strains that used an Affymetrix GeneChip designed from the closely related *V*. *parahaemolyticus* RIMD2210633 genome sequence. Overall there was excellent correlation between the microarray and RNA-Seq data. Eleven transcription factors under OpaR control were identified by both methods and further confirmed by quantitative reverse transcription PCR (qRT-PCR) analysis. Nine of these transcription factors were demonstrated to be direct OpaR targets via *in vitro* electrophoretic mobility shift assays with purified hexahistidine-tagged OpaR. Identification of the direct and indirect targets of OpaR, including small RNAs, will enable the construction of a network map of regulatory interactions important for the switch between the nonpathogenic and pathogenic states.

## Introduction

*Vibrio parahaemolyticus* is a marine bacterium that causes gastroenteritis in humans when consumed while eating raw or undercooked shellfish and potential life-threatening systemic infections if it enters open wounds [1]. It has the ability to survive in a variety of environments with varying ranges of salt and temperature [2] and in particular, oyster-associated outbreaks are occurring with increasing frequency worldwide [3]. In the USA, it is an issue throughout the Atlantic, Gulf and Pacific coasts [4,5]. BB22OP [6], an environmental non-clinical isolate, is one of the most genetically characterized *V*. *parahaemolyticus* strains. It exhibits a profound phenotypic change from opaque (OP) to translucent (TR) when OpaR, a key transcriptional regulator involved in quorum sensing (QS), is mutated [7]. During bacterial QS, intercellular signaling molecules enable individual cells to function in unison to coordinately control outputs such as biofilm formation, antibiotic production, bioluminescence, and virulence factor expression. Signaling molecules, called autoinducers (AIs), are produced and released by the bacteria and once a critical concentration of signal is achieved the bacteria respond by changing their patterns of gene expression [8–12]. Different bacterial species produce diverse AI molecules and use these communication signals to control specific traits. Defining the QS mechanisms specific to *V*. *parahaemolyticus* BB22OP will provide a better understanding for how colonization and pathogenicity are coordinately regulated as the organism transitions between environmental and host-associated niches.

OpaR is a homologue of LuxR in *Vibrio harveyi* [7]. It is the master QS regulator that controls opacity, biofilm formation, swarming motility, type III secretion, and type VI secretion systems in *V*. *parahaemolyticus* [13]. The QS pathway upstream of OpaR in *V*. *parahaemolyticus* appears to utilize a phosphorelay cascade similar to that of the well-studied *V*. *harveyi* system [13]. In the *V*. *harveyi* QS system, at low cell density, which corresponds to low levels of three different AIs, LuxU is phosphorylated by upstream receptors that sense the AIs. LuxU-P in turn controls the phosphorylation state of the sigma^54^- dependent regulator, LuxO [14]. Then the activated LuxO-P induces the expression of five small quorum regulatory RNAs (Qrrs) that target *luxR* transcripts, silencing their translation. At high cell density, the higher concentration of AIs causes LuxO dephosphorylation, the Qrrs are not expressed, and LuxR is synthesized [15].

In *V*. *parahaemolyticus*, when its LuxR homolog OpaR is not synthesized, this strain produces translucent colonies, is swarm proficient and expresses virulence factors such as the type III secretion system on chromosome 1 (T3SS1). Conversely, when OpaR is present, the *V*. *parahaemolyticus* colonies are opaque. The OpaR-producing strain swarms poorly and is less virulent in cytotoxicity assays [13]. *V*. *parahaemolyticus* BB22OP is of particular interest because it has a functional QS system, whereas *V*. *parahaemolyticus* RIMD2210633 and many other clinical isolates are postulated to have mutations in genes within the QS cascade that lock the system in the pathogenic state, e.g., mutations resulting in constitutively active variants of LuxO that cause repressed production of OpaR at all cell densities [13].

Previous microarray work comparing the transcriptome of *V*. *parahaemolyticus* BB22OP (LM5312) to an *opaR* deletion strain BB22TR (LM5674) was performed using an Affymetrix GeneChip designed using the *V*. *parahaemolyticus* RIMD2210633 genome [13]. This microarray analysis provided valuable insights into the QS regulon. However, the recently completed assembly of the BB22OP genome has revealed a number of differences between RIMD2210633 and BB22OP [16], including genes unique to RIMD2210633 and genes unique to BB22OP. Further analysis of BB22OP using RNA-Seq [17,18] as a technique to capture the entire transcriptome, is now providing a more complete understanding of the QS pathway for the BB22OP strain, as well as information with respect to operon structure, transcription start and stop sites, and small noncoding RNAs expression.

In this study, RNA-Seq was used to compare the transcriptome of *V*. *parahaemolyticus* BB22OP (*opaR* ^+^, LM5312) with BB22TR (∆*opaR1*, LM5674), at one time point during mid-exponential phase growth. Overall there was excellent agreement between the microarray and RNA-Seq data. Hundreds of genes were found to be up or down regulated by OpaR. To begin to trace the network of direct and indirect QS regulation, eleven transcription factors regulated greater than four-fold by OpaR were identified and their QS control was further confirmed by quantitative reverse transcription PCR (qRT-PCR) analysis. The transcriptional start sites for the genes encoding these transcription factors were located using the RNA-Seq data and direct OpaR binding to their promoters was analyzed by electrophoretic mobility shift assays (EMSAs). In addition, the differential expression of previously predicted small noncoding RNAs was examined. Collectively, these efforts have further defined the QS signaling network downstream of OpaR in *V*. *parahaemolyticus*.

## Materials and Methods

### Growth conditions

The strains used for these experiments have been described [13]; *V*. *parahaemolyticus* BB22OP strain LM5312 is *opaR* ^*+*^ and BB22TR strain LM5674 contains the ∆*opaR1* allele, which is an 85-bp deletion in the upstream and N-terminal coding region of *opaR*. Extraction of total RNA following, growth and harvesting procedures, were performed similarly to Gode-Potratz et al [13]. Heart infusion agar (HI, 25g/L, Remel, Lenexa, KS with 15g/L NaCl and 2% agar) was used to grow LM5312 and LM5674 overnight. The cells were then scraped from the agar plates and suspended in HI broth to an OD_600_ of 0.05. Fifty microliters of the suspended cells were spread on fresh HI agar and incubated at 30°C for 6 hours. After incubation, the cells were again scraped from the agar and suspended to an OD_600_ of 1.0 in RNAprotect (Qiagen, Gaithersburg, MD) that had been diluted 2-fold in phosphate-buffered saline (8 g NaCl, 0.2 g KCl, 1.44 g Na_2_HPO_4_, and 0.24 g KH_2_PO_4_ per liter), pH 7.1. Cells were vortexed for 10 sec and incubated at room temperature for 5 min to stabilize mRNA before samples were centrifuged for 10 min at 5000 x *g*. Cell pellets were stored at -20°C until RNA extraction was completed.

### RNA extraction

Cell pellets were suspended with 200 μl of Tris EDTA (TE) buffer (pH 7.0) containing 15 mg/ml lysozyme (Sigma, St. Louis, MO), 20 μl proteinase K (Qiagen), and 700 μl of QIAzol (Qiagen). The total RNA was harvested using a miRNeasy kit (Qiagen) following the bench protocol in the miRNeasy Mini Handbook. The sample purity and concentration were first analyzed using a NanoPhotometer (Implen, Westlake Village, CA) and the final quantity and quality of total RNA was assessed on an Agilent Bioanalyzer 2100 at the Virginia Bioinformatics Institute (VBI) (Blacksburg, VA). Total RNA was subjected to a depletion protocol to enrich the mRNA fraction using the Ribo-Zero rRNA Removal Kit for Gram-negative Bacteria (Epicentre, Madison WI). The rRNA-depleted sample was assessed for quality, including rRNA removal, prior to sample processing for Illumina sequencing (VBI) with single-paired end 50-bp reads.

### ExpressSeq pipeline for RNA-Seq data analysis

The *V*. *parahaemolyticus* LM5312 (*opaR* ^+^) and LM5674 (∆*opaR1*) RNA-Seq data were processed as per Ramachandran et al. [19] with the transcriptome sequencing read files in FASTQ format transformed into FASTA format using the SeqIO module of BioPython [20]. Then the standalone BLAST+ suite [21,22] was used to align the reads to the annotated *V*. *parahaemolyticus* BB22OP protein coding sequences downloaded from the NCBI nucleotide pages for Chromosome 1 (CP003972.1) and Chromosome 2 (CP003973.1). First, a BLAST database and its index were created using the FASTA files for each of the samples using the makeblastdb and makembindex BLAST+ routines. Second, for each sample, standalone blastn queries were performed using the nucleotide sequences for the *V*. *parahaemolyticus* BB22OP protein coding genes against the BLAST database for each sample. In blastn a word size of 16 and a maximum e-value of 1.e-10 were chosen for alignment. Finally, the table format output (-outfmt 6) of each blastn query was subsequently processed using an awk-based shell script to count and list the total number of blastn hits for each of the protein-coding genes in the *V*. *parahaemolyticus* data set. Although the BLAST alignment is slower than other alignment algorithms (such as Burrows-Wheeler Aligner [23]), every step uses analysis tools that can repeatedly be used for each protein coding gene and for any other transcribed sequence [24]. Since read hits are counted only if the alignment to the reference sequences are sufficiently close (e-value < 1.e-10), no quality filtering is required and no biases are introduced by preprocessing the reads using quality scores. The one caveat with this pipeline is that reads can be counted as hits to multiple genes, so that differential expression levels for paralogous genes with sufficient sequence similarity will be difficult to determine without further analysis.

Subsequent data processing was done using Microsoft Excel. To calculate the fold-changes in differential expression, the read counts for each sample were first normalized to the total number of reads mapped to all genes to determine the numbers of reads aligned to each gene per million mapped reads (RPM). Then the fold-change for each gene was computed by taking the ratio of the RPM values for the two samples. Finally, the error for the ratios of normalized gene expression levels were conservatively estimated using the standard deviation of the ratios across all genes with fold changes < 4-fold on Chromosome 1 (94% of the genes) and Chromosome 2 (91% of the genes). This error estimate assumes that the expression of most genes is not changed and the overall difference in expression levels measured between the two samples is a good surrogate for biological replicates and that a multiplicative biological/sample preparation dominates over the purely technical sampling errors [24].

### Procedure for identifying OpaR regulon genes of interest

Previous microarray data for the *V*. *parahaemolyticus* BB22OP LM5312 (*opaR* ^*+*^ *)* and BB22TR LM5674 (∆*opaR1)* strains generated using an Affymetrix GeneChip designed to the *V*. *parahaemolyticus* RIMD2210633 strain [25] was initially analyzed for transcriptional regulators differentially expressed four-fold or more. Then the RNA-Seq data was also analyzed to expand the list to include any additional regulators that exhibited a similar four fold or greater change in expression. The normalized RPM for the LM5312 (*opaR* ^*+*^ *)* and LM5674 (Δ*opaR1)* strains were compared to determine the regulation. Genes that were activated by OpaR had a greater RPM in the wild-type strain and genes that were repressed by OpaR had a greater RPM in the deletion strain. The list of regulated genes was also filtered to remove low expression genes with ~ 1x read coverage or less (corresponding to an absolute read alignment count of 50). In addition, genes that are unique to BB22OP and not found in RIMD2210633 were examined for differential expression to determine the information that was missed by using the *V*. *parahaemolyticus* RIMD2210633 strain GeneChip in the original microarray study [13].

### Quantitative reverse-transcription PCR (qRT-PCR)

Cloning primers were designed and used to amplify the coding regions of 11 transcription factors of interest identified as being differentially expressed greater than four-fold in the microarray and/or RNA-Seq studies, plus two negative control genes *rpoD and fliA*_*P*_ (for polar flagella), and the 16S rRNA gene for normalization of data (S1 Table). PCR was performed using Phusion polymerase (New England Biolabs (NEB), Ipswich, MA). The annealing temperatures used were gene specific and are listed in S1 Table. Following PCR amplification, the coding regions for the 14 genes were ligated into pGEM-T vector (Promega, Madison, WI), transformed into *E*. *coli* Top 10 cells and the recombinant plasmids were sequenced at VBI to confirm sequence fidelity.

Primer Express 3.0 (Applied Biosystems) was used to design primer pairs for qRT-PCR analysis of the 14 genes (S1 Table). Primer design parameters were set as: 18–24 base pairs, a melting temperature (Tm) of 60–65°C and amplicon length of 80–120 bp. Plasmid DNA was used as template to optimize qRT-PCR primers to within 95% and 105% efficiency, at concentrations ranging from 0.001 ng to 1 ng per 25 μL reaction. The reaction mix contained 300 nM each of the specific forward and reverse primer pair and 2x SYBR green PCR master mix (Applied Biosystems) diluted to a 1x concentration with dH_2_O and template DNA or cDNA.

*V*. *parahaemolyticus* LM5312 (*opaR* ^*+*^) and LM5674 (∆*opaR1*) were grown and RNA was extracted as described above for the RNA-Seq method [13]. The RNA was then converted to cDNA using the High Capacity cDNA Reverse Transcription Kit (Applied Biosystems, Foster City, CA) per the manufacturer’s instructions. The cDNA was quantified using a NanoPhotometer and samples were used as templates in an Applied Biosystems 7300 Real-Time PCR system. cDNA was used at a concentration of 10 ng per 25 μL reaction containing 300 nM each of the specific forward and reverse primer pair and 2x SYBR green PCR master mix (Applied Biosystems) diluted to a 1x concentration with dH_2_O. Reactions were carried out in triplicate in a MicroAmp Optical 96-well Reaction Plate (Applied Biosystems). The thermal cycler settings were programmed for 95°C for 10 min, 45 cycles of 95°C for 15 sec and the appropriate annealing temperature (S1 Table) for 1 min. The annealing step was also set as the data collection point. A dissociation stage was added at the end of the PCR run to confirm specific product amplification. The relative expression of the genes of interest was then determined using the PFAFFL method [26] and standardized using 16S rRNA relative expression. The error in fold changes for each gene was estimated by computing the standard deviation for four replicates of the qRT-PCR measurements.

### OpaR protein purification

An N-terminally 6x-His-tagged OpaR fusion protein gene was constructed in the pET28a vector and purified following the procedure previous outlined by Zhang et al. [27]. Functionality of the his-tagged protein was confirmed by EMSAs.

### EMSAs

A published master QS regulator binding site (MQSR) matrix [27] was used with the position-specific scoring matrix (PSSM) program, PATSER (Regulatory Sequence Analysis Tool) [28], to identify putative OpaR-binding sites in the regions upstream of the 11 transcription factors of interest (S2 Table). Transcription start sites were determined by viewing the alignment of reads to the genome in the Seqman Pro (Lasergene, DNASTAR Inc., Madison, WI) browser and looking for a sharp cliff of at least ten reads in height upstream from the translation start site. A candidate promoter region was designated from the end of the adjacent upstream gene, to the +1 site of the gene of interest. These promoter regions were cloned via PCR amplification using primers designed with a FAM label at the end closest to the +1 site (S2 Table). The fragment was visualized using 1% agarose gel electrophoresis, purified using the Qiaquick Gel Extraction kit (Qiagen) and quantified using a NanoPhotometer.

Purified His-OpaR in the concentration range of 0–400 nM, was used to test binding to the putative promoters in reactions with 10 nM FAM-DNA and EMSA buffer (1 mM MgCl_2_, 0.5 mM EDTA, 0.5 mM DTT, 50 mM NaCl, 10 mM Tris-HCl [pH7.5], 0.05 mg/ml poly d(I-C), 150 μg/ml of BSA) [27]. A control competition reaction with 100 nM unlabeled DNA and 100 nM OpaR was performed to prove specificity of the binding. The reaction was visualized on a 4%, 5%, or 6% TBE (10.8 g/L Tris, 5.5 g/L boric acid, 4 ml 0.5M EDTA) polyacrylamide gel depending on the size of the fragment [27] (S2 Table).

### Putative small RNA (sRNA) identification

Putative sRNAs were identified using the sRNAPredict 2 [29] and BSRD [30] prediction databases, which self-contained the RIMD2210633 data for analysis. This cumulative list was then analyzed to determine the subset of sRNAs identified in both databases. The RNA-Seq expression of the sRNAs from this shortened list was determined as previously described above for the protein coding genes. In addition, previously unidentified sRNAs were located using the intergenic regions of microarray data [13] plus an examination of the RNA-Seq data to determine if the sRNA was real or part of an untranslated region of a neighboring gene. Expression of one novel sRNA that was controlled by OpaR and not previously predicted or deposited in a database was verified using qRT-PCR as described above for the transcriptional regulators.

### Accession numbers for data

The read data for the *V*. *parahaemolyticus* BB22OP LM5312 (*opaR* ^*+*^ *)* QS-proficient strain, and *V*. *parahaemolyticus* BB22TR LM5674 (∆*opaR1*) QS-deficient strain, have been deposited in the NCBI Sequence Read Archive (SRA) with accession numbers GSM1297676 and GSM1297677, respectively. An Excel file summarizing the differential gene expression in total counts and normalized RPM, using the BB22OP and RIMD2210633 annotations, has been deposited in the NCBI Gene Expression Omnibus (GEO) database (GEO Accession GSE53639).

## Results

### Overview of RNA-Seq data analysis of the OpaR regulon in *V*. *parahaemolyticus*

**Fig 1:**
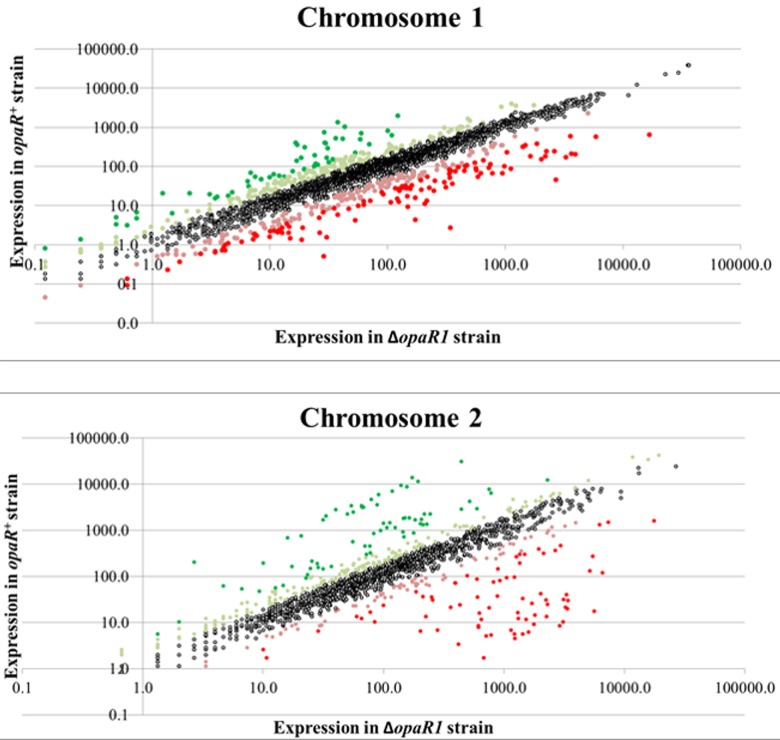
Differential mRNA expression controlled by OpaR in *V*. *parahaemolyticus*. Graphical representation of the RNA-Seq data from *V*. *parahaemolyticus* BB22OP *s*train LM5312 (*opaR* ^*+*^) and BB22TR strain LM5674 (∆*opaR1*) for Chromosome 1 (Panel A) and Chromosome 2 (Panel B). The RPM change of normalized expression for most genes (unfilled circles) falls around a line with a slope of 1, indicating that they are equally expressed in both strains. Points with lighter tone are controlled by OpaR greater than 2-fold and the darker points are controlled by OpaR greater than 4-fold with green shades highlighting activated genes and red shades highlighting repressed genes.

A screening RNA-Seq analysis established the full scope of the *V*. *parahaemolyticus* OpaR regulon by comparing the transcriptome of *V*. *parahaemolyticus* LM5312 (*opaR* ^*+*^) and LM5674 (∆*opaR1)*. The total RNA extracted from LM5312 and LM5674 had RNA integrity values (RIN) of 9.8 and 10 respectively. Following rRNA depletion, approximately 30 million 50-bp transcripts were obtained for each sample corresponding to an average gene coverage of more than 300 times. Greater than 15% of the genome showed a two-fold or greater change in expression between the *opaR* ^+^ and ∆*opaR1* samples (Fig 1). Similar to what had been observed in the microarray comparison [13], OpaR activated genes involved in capsular polysaccharide production and the type VI secretion system located on chromosome 2 (T6SS2), and repressed the type III secretion system 1 located on chromosome 1 (T3SS1), type VI secretion system 1 (T6SS1), and the lateral flagellar regulon. Chromosome 1 has a total of 2848 predicted genes, of these 237 were activated two-fold or greater by OpaR and 219 were repressed at least two-fold. Chromosome 2 has 1600 potential genes, 206 genes were activated and 128 genes were repressed two-fold or more. The RNA-Seq data confirmed that OpaR plays a significant role in global regulation of gene expression in *V*. *parahaemolyticus* BB22OP as had been previously demonstrated via microarrays using an Affymetrix GeneChip designed to the *V*. *parahaemolyticus* RIMD2210633 strain [25]. The microarray data and RNA-Seq data showed a high degree of similarity (S1 Fig). Only 18 genes appeared to exhibit expression changes in opposite directions for these two platforms (S3 Table). Since six of the discordant genes from chromosome 2 were phosphate transport related and two of the discordant genes from chromosome 1 were DNA uptake related genes, it is likely that most of these minor differences in expression may be attributed to subtle differences in growth conditions.

Genes that are unique to *V*. *parahaemolyticus* BB22OP and not found in *V*. *parahaemolyticus* RIMD2210633 were also analyzed to determine what limitations had been imposed on the BB22OP transcriptome analysis by using the RIMD2210633 GeneChip. Twenty-four genes that were regulated four-fold or greater by OpaR and had greater than 50 reads for at least one of the sequencing reactions were identified as being unique to BB22OP (S4 Table). Three of these genes are on chromosome 2 and 21 are on chromosome 1. Of the 21 unique OpaR-regulated genes on chromosome 1, fourteen are found in an integron region that is unique to the BB22OP strain [16]. Twenty-one of the 24 unique genes are annotated as hypothetical proteins, whereas the other three genes encode a putative lipase, an acetyltransferase and a halogenase.

NCBI BLAST was used to analyze the homology of 21 genes encoding hypothetical proteins to determine if a function could be assigned. VPBB_1425 shares 74 of its 132 base pairs with the gene for an Rhs-family protein found in *V*. *parahaemolyticus* UCM-V493 with the locus tag VPUCM_1621. However, the Rhs-family protein in UCM-V493 is 960 bp suggesting that VPBB_1425 may be truncated. VPBB_2301 matches 116 bp of its 117 bp to a 117 bp gene encoding glutamate-1-semialdehyde aminotransferase in *V*. *parahaemolyticus* O1:Kuk str. FDA_R31 with the locus tag M634_14765. This suggests that VPBB_2301 should also be annotated as encoding glutamate-1-semialdehyde aminotransferase.

### Identification of transcriptional regulators in the OpaR regulon and validation of their expression via qRT-PCR

The RNA-Seq data (S4 Table) and microarray data (previously published [13]) were analyzed for OpaR-controlled transcription factors that were differentially expressed four fold or greater between the LM5312 (*opaR* ^*+*^) and LM5674 (∆∆*opaR1)* strains, using at least one of the two approaches. Eleven transcription factors were chosen for further study as described above (Table 1) and qRT-PCR was performed to validate the changes in gene expression observed in the Illumina sequencing. Two separate RNA samples extracted independently of the sample generated for RNA-Seq analysis were used. The expression of the 16S rRNA coding region was used as reference to normalize expression of other genes. Although the ratios of change in expression obtained via RNA-Seq and qRT-PCR differed slightly, all genes displayed similar trends in regulation (activation vs. repression) (Table 1). The two control genes, *rpoD* and *fliA*_P_, had been previously demonstrated to be constitutively expressed in *V*. *parahaemolyticus* BB22OP regardless of OpaR function [13].

**Table 1:** Validation of Transcription Factors Highly Regulated by OpaR via qRT-PCR.

BB22OP ID	RIMD 2210633 ID	Annotation	Mode of OpaR Regulation	Microarray Data^a^	*opaR* ^*+*^/Δ*opaR1* RNA-Seq^b^	*opaR* ^*+*^/Δ*opaR1* qRT-PCR^c^
Chromosome 1						
VPBB_0491	VP0514	*cpsR*	activated	3.10 ± 0.31	5.76	12.87 ± 5.86
VPBB_0645	VP0675	*crl* family	activated	4.91 ± 0.27	1.98	3.50 ± 1.37
VPBB_2530	VP2710	*csgD/vpsT* family	activated	6.06 ± 0.94	2.45	2.88 ± 1.05
VPBB_1307	VP1391	*fhlA*	repressed	0.03 ± 0.01	0.06	0.31 ± 0.13
VPBB_1322	VP1407	*asnC* family	repressed	0.11 ± 0.01	0.32	0.12 ± 0.03
VPBB_1558	VP1699	*exsA*	repressed	0.15 ± 0.02	0.29	0.30 ± 0.16
VPBB_2619	VP2762	*aphA*	repressed	0.24 ± 0.02	0.20	0.26 ± 0.07
Chromosome 2						
VPBB_A0554	VPA0606	*araC* family	activated	5.12 ± 0.57	6.12	3.43 ± 0.90
VPBB_A0869	VPA0947	*arsR* family	activated	4.38 ± 0.65	8.05	3.20 ± 1.74
VPBB_A1319	VPA1446	*cpsQ*	activated	3.54 ± 0.24	8.14	3.62 ± 0.19
VPBB_A1405	VPA1538	*lafK*	repressed	0.02 ± 0.003	0.01	0.01 ± 0.01
Controls						
VPBB_0387	VP0404	*rpoD*	none	0.99 ± 0.02	0.93	0.65 ± 0.10
VPBB_2050	VP2232	*fliA*_P_ (Polar)	none	0.39 ± 0.25	0.50	0.75 ± 0.19

^a^For the microarray data, the standard deviation of the normalized expression in four biological replicates of the ∆*opaR1* strain was compared to three biological replicates of *opaR* ^*+*^ strains.

^b^ RNA-Seq data is fold change of the *opaR* ^*+*^ strain gene expression divided by the ∆*opaR1* strain gene expression. Error for the ratios of normalized gene expression levels were conservatively estimated using the standard deviation ratios across the majority of genes with less than 4-fold change. The standard deviation for chromosome 1 is 1.53 and the error for chromosome 2 is 1.59.

^c^ The error in fold changes for each gene was estimated by computing the standard deviation for four replicates of the qRT-PCR measurements.

### EMSA analysis of OpaR-controlled transcription factors

**Fig 2:**
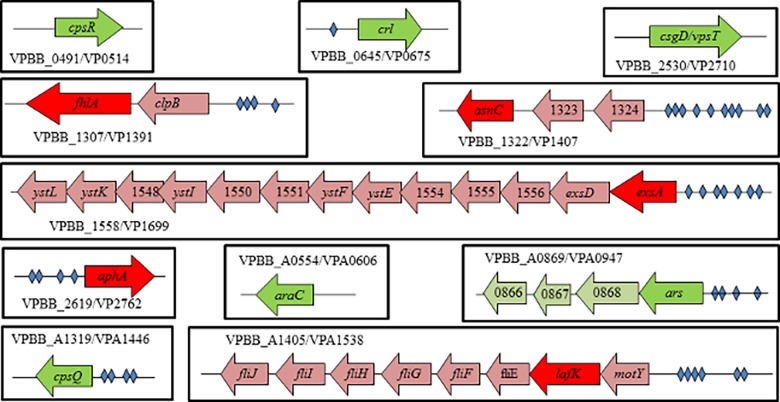
Organization of select gene loci of *V*. *parahaemolyticus* in the OpaR regulon. Cartoon gene maps, not to scale, are provided for the promoter regions and gene organization near OpaR-regulated transcription factors identified through the RNA-Seq data analysis. Dark green arrows represent genes of transcription factors activated by OpaR, dark red arrows indicate genes of repressed transcription factors and the lighter arrows illustrate other genes in the operons of interest. The diamonds represent predicted OpaR-binding sites from PATSER data (see Table 2 for specific locations and scores).

A bioinformatics search using the PATSER program [28] and a previously published LuxR family binding sequence [27] identified putative binding sites upstream of eight of the 11 genes encoding transcription factors (Table 2). Two of the genes, VPBB_1558 (encoding the T3SS1 regulator ExsA) and VPBB_A0869 (encoding an ArsR-family regulator), appeared to be at the beginning of operons with predicted OpaR-binding sites directly upstream of the transcription factor; whereas three of the regulatory genes, VPBB_1307 (encoding an FhlA-family transcription factor), VPBB_1322 (encoding an AsnC-family regulator), and VPBB_A1405 (encoding the flagellar regulator LafK), are located within operons with the potential OpaR-binding site not directly upstream of the gene encoding the transcription factor (Fig 2). Three genes, VPBB_0645 (encoding a Crl-family regulator), VPBB_2619 (encoding the low-cell density regulator AphA), and VPBB_A1319 (encoding the capsule regulator CpsQ), are not part of an operon and have the putative binding site directly upstream of the start site. It was predicted that OpaR would not bind the promoter regions of VPBB_0491(encoding another capsule regulator CpsR), VPBB_2530 (encoding a CsgD/VpsT-family regulator), or VPBB_A0554 (encoding an AraC-family regulator) because they lacked potential binding sites. Most of the PATSER scores for the eight candidate binding sites were between three and five (Table 2). Only three transcription factor genes, *aphA*, *exsA*, and *lafK*, had upstream putative binding sites for OpaR with PATSER scores greater than seven, which is the default setting for significance in PATSER.

**Table 2:** Predicted OpaR-binding sites in Promoters of Select Transcription Factors^a^.

Transcription factor: BB22OP (RIMD2210633)/ annotation	Score	OpaR-binding site 5′ end	OpaR-binding site 3′ end	Sequence (5′ to 3′)^e^
**VPBB_0645 (VP0675) *crl* family**	3.73	−57	−38	AAAAAATAATAATCATTATT
**VPBB_1307 (VP1391) *fhlA* family** ^**b**^	4.39	−296	−277	TATTGGATAAAAAACCAATA
	5	−263	−244	TAATAATAAATTGATTCAAT
	3.34	−259	−240	AATAAATTGATTCAATTTTA
	4.74	−255	−236	AATTGATTCAATTTTAAAAA
**VPBB_1322 (VP1407) *asnC* family** ^**c**^	3.48	−397	−378	AACTATCAAAATAGGCATTT
	3.26	−388	−369	AATAGGCATTTCAACCATTA
	3.72	−287	−268	TAATAAAAAAACACCTAATA
	4.14	−272	−253	TAATATTAAGTTTATTAAAA
	5.06	−264	−245	AGTTTATTAAAATGTTCATT
	6.35	−182	−163	AATTGATATTAATATTAGGA
	5.59	−99	−80	TTTTATCTATAAAATCAGAA
	5.9	−40	−21	AATAAAAAAATCTATCTTTA
	3.69	−26	−7	TCTTTATTATTTTATTATCA
	5.37	−23	−4	TTATTATTTTATTATCAACT
**VPBB_1558 (VP1699) *exsA***	3.38	−369	−350	TAATTTTTATTATCATATTA
	3.89	−366	−347	TTTTTATTATCATATTAGTA
	6.71	−362	−343	TATTATCATATTAGTAATTT
	7.97	−354	−335	TATTAGTAATTTAATTTATT
	4.47	−342	−323	AATTTATTCTAATATAAAAA
	5.74	−283	−264	AATAGACTTATAAGAAAGTA
	3.92	−79	−60	AAATAACAACAAACAAAGTA
**VPBB_2619 (VP2762) *aphA***	3.54	−299	−280	TATTAACTACAAAATAACTC
	10.35	−279	−260	TATTGAGTATTATGTTAGTT
	4.06	−94	−75	TACTTATACATTAAAAAACT
	3.81	−23	−4	TATTGACCATTTGGATTGAA
**VPBB_A0869 (VPA0947) *arsR* family**	5.59	−123	−104	TATAATTAACTAAATTAGAA
	5.58	−90	−71	ATTTATCTAAATTCACAATT
	4.63	−47	−28	TATTGTTTTAGTAATATCTA
	3.53	−39	−20	TAGTAATATCTAATTTAGTT
**VPBB_A1319 (VPA1446) *cpsQ***	4	−133	−114	TTTTGTTTCTGTTTTTAATA
	5.26	−121	−102	TTTTAATAAATTAGTAATTC
	3.11	−46	−27	TGCTGATAATTAACTACGAA
	4.06	−39	−20	AATTAACTACGAAATTAAGA
**VPBB_A1405 (VPA1538) *lafK*** ^**d**^	4.89	−214	−195	CATTTATAAATCGGTTAATA
	3.63	−198	−179	AATAAATTAAAATAGCAACT
	7.26	−122	−103	TTATTATAAAAAAATTAATA
	3.91	−118	−99	TATAAAAAAATTAATATAAA
	4.21	−116	−97	TAAAAAAATTAATATAAATA
	4.76	−113	−94	AAAAATTAATATAAATAGTT
	3.59	−110	−91	AATTAATATAAATAGTTGTT

^a^Putative binding sites identified through PATSER using MQSR matrix [27]

^b^Scores and locations are upstream of VPBB_1307 (VP1392), the first gene of the operon.

^c^ Scores and locations are upstream of VPBB_1322 (VP1409), the first gene of the operon.

^**d**^ Scores and locations are upstream of VPBB_A1405 (VPA1538), the first gene of the operon.

**Fig 3:**
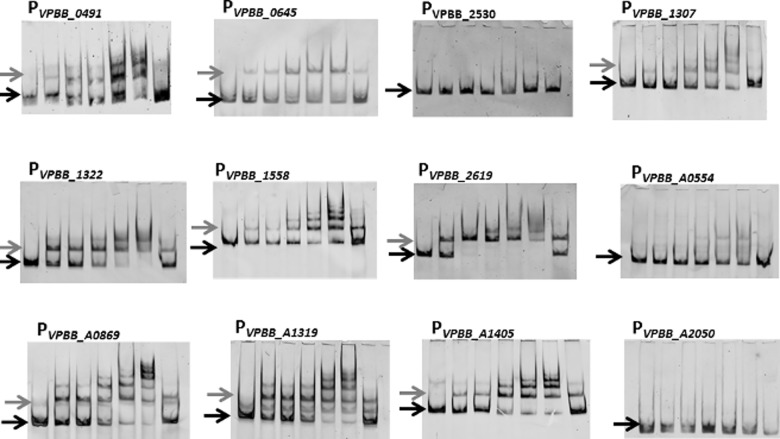
EMSA analysis of putative OpaR direct targets. Each panel is labeled with the promoter being analyzed. The black arrow indicates unbound DNA and the grey arrow indicates the DNA shift due to bound OpaR, with additional complexes with higher mobility present at some promoters. The concentration of FAM-labeled DNA probe in all lanes is 10 nM. The lanes within each panel (left to right) consist of the following: DNA probe with 0 nM, 25 nM, 50 nM, 100 nM OpaR, 200 nM or 400 nM OpaR. The sample in the far right lane contains DNA probe plus 100 nM of OpaR with 100 nM unlabeled DNA for competition. Gene names are: VPBB_0491(*cpsR)*, VPBB_0645 (*crl* family*)*, VPBB_2530 (*cspD/vpsT* family), VPBB_1307 *(fhlA* family*)*, VPBB_1322 *(asnC* family*)*, VPBB_1558 *(exsA)*, VPBB_2619 *(aphA)*, VPBB_A0554 (*araC* family), VPBB_A0869 *(ars* family*)*, VPBB_A1319 *(cpsQ* family*)*, and VPBB_A1405 (*lafK)*.

Remarkably, the EMSA analysis demonstrated that OpaR bound nine of the 11 target promoters (Fig 3). Thus, the weaker scoring OpaR-binding sites appear to be functional. This was clearly illustrated by the multiple shifted bands observed in several of the promoters. The MQSR matrix [27] utilized with PATSER, in combination with the RNA-Seq data selecting for highly regulated genes, appeared to be quite effective in identifying possible OpaR-binding sites since even sequences with low scores appeared to be valid based on the EMSA results. OpaR did not bind the promoter region of VPBB_2530 and VPBB_A0554 (Fig 3), which were two of the three genes that lacked predicted binding boxes. However, it did bind to the promoter of VPBB_0491 with no predicted OpaR-binding site, illustrating that there are limits to the ability of the bioinformatics tools to identify all possible OpaR targets. This in part may be due to the fact that the MQSR matrix is not specific for OpaR, since binding sites for multiple LuxR homologues were used to design it. Nevertheless, for the purpose of this study, the MQSR matrix did serve as a useful tool to identify putative target genes for further EMSA analysis.

### Bioinformatics analysis of RNA-Seq data to identify additional OpaR direct targets

Since the bioinformatics method was quite effective in identifying putative OpaR targets, a second purely theoretical bioinformatics approach was applied to the RNA-Seq data in an effort to identify more OpaR direct targets. First, a list of genes with a five-fold or greater change in OpaR-dependent expression was generated. The more stringent five-fold cut off was used to help decrease possible false positives. This initial list was then cross-referenced with 2702 putative OpaR-binding sites with a PATSER score of 3 or higher identified by the PATSER program using the MQSR matrix with the RIMD2210633 genome [31]. The RIMD2210633 genome was utilized since it was already accessible in the PATSER program and there were only five unique BB22OP genes with greater than five-fold differential OpaR-dependent expression. This analysis yielded a list that included eight of the 11 transcription factors of interest and 60 additional OpaR-controlled genes that may have OpaR-binding sites in the *V*. *parahaemolyticus* BB22OP genome (S5 Table).

The 60 newly identified genes, not including the eight transcription factors, can be classified into nine different categories. Sixteen genes appear to encode hypothetical proteins, nine encode methyl-accepting chemotaxis related proteins, seven encode cell surface related proteins, seven encode transporter proteins, seven encode regulatory or metabolic enzymes, three genes encode lateral flagellar proteins, five encode proteins with domains predicted to be involved in c-di-GMP synthesis or degradation, and one gene encodes the polar flagellar protein FlhA. There are five remaining genes that do not classify into one of the above categories: VPBB_1560 (encoding type III secretion regulator ExsC), VPBB_1309 (encoding type VI secretion Hcp protein), VPBB_1336 (encoding SM-20-related protein), VPBB_1851 (encoding cytochrome c4), and VPBB_2845 (encoding putative signal peptide protein). While further analysis of these putative direct targets of OpaR was beyond the scope of this study, their identification suggests that OpaR may directly control a variety of genes in *V*. *parahaemolyticus*.

After the BB22OP genome sequence became available in the PATSER database, an analysis of the promoter regions of the five unique BB22OP genes regulated five-fold or more by OpaR was similarly performed. Using these criterion, results indicated only one gene specific to BB22OP as being theoretically directly regulated by OpaR. VPBB_A0413 is a gene encoding a hypothetical protein with a PATSER score of 7.8 and *opaR* ^*+*^/Δ*opaR1* RNA-Seq regulation of 0.11. The predicted OpaR binding site is -412 to -393 upstream of the start site (S5 Table).

### Bioinformatics analysis of RNA-Seq data to identify putative sRNAs

Outputs from two different published databases, BSRD [30] and sRNApredict2 [29], that utilized the *V*. *parahaemolyticus* RIMD2210633 genome, were initially compared to help identify sRNAs predicted to also exist in BB22OP. For Chromosome 1, sRNApredict2 predicted 50 candidate sRNAs, BSRD predicted 45, but only 14 were predicted by both programs. The sRNApredict2 program identified 42 possible sRNAs for chromosome 2, while BSRD predicted 20, with only six predicted by both programs. Both programs successfully predicted the five Qrrs believed to control OpaR translation [29,30].

The BB22OP RNA-Seq data validated expression of several predicted sRNAs described above in at least one of the two strains examined. RPM values were computed for each of the predicted sRNAs sequences from the two RNA-Seq samples, revealing six predicted sRNAs found in one or both of the sRNA databases that were differentially regulated greater than four-fold by OpaR (Table 3). Four of the six sRNAs were too small for efficient qRT-PCR validation, and since all of them were already deposited into public databases, no additional analysis was performed. The BB22OP RNA-Seq data was also analyzed for differential expression of novel sRNAs not predicted by the two databases by looking for the pileup of reads mapping to intergenic regions. Two candidates were discovered to be possible OpaR controlled sRNAs. One appears to be a 5’ untranslated region upstream of gene VPBB_1422 rather than a distinct sRNA. VPBB_1422 (VP1517) encodes an Rhs-family protein; its upstream region was similarly regulated in the microarray analysis (~34-fold). The second candidate has all the characteristics of a sRNA. This putative sRNA, called Srr, is located between genes VPBB_1217 and VPBB_1218 on the VP B22OP chromosome 1. The Srr RNA was previously identified as being part of the lateral flagellar regulon and was repressed 73-fold by OpaR [13,25]. The RNA-Seq data showed Srr as being repressed 222.69 ± 1.54 fold by OpaR (Table 3). The existence of this novel sRNA was verified, and OpaR-control of its expression was validated when subsequent qRT-PCR analysis demonstrated 167.93 ± 62.92-fold repression by OpaR. Thus, along with the transcripts of the lateral flagellar genes, it is one of the most highly regulated transcripts in the entire OpaR regulon.

**Table 3:** Putative sRNAs and 5’-untranslated leaders regulated four-fold or more by OpaR.

sRNA ID	Flanking BB22OP genes	Length (bp)	Mode of OpaR Regulation	*opaR* ^+^/Δ*opaR1* RNA-Seq^f^
**Chromosome 1:**				
Candidate^a^ 18	VPBB_2071-VPBB_2072	93^d^	repressed	0.15
Candidate^a^ 33	VPBB_2279-VPBB_2280	133^d^	repressed	0.15
Srr (swarm-specific RNA)^b^	VPBB_1217-VPBB_1218	190^e^	repressed	0.005
**Chromosome 2:**				
Cyclic di-GMP-II Riboswitch^c^	VPBB_A1460-VPBB_A1461	105^d^	repressed	0.07
MicX^c^	VPBB_A1271-VPBB_A1272	196	activated	4.58
Purine_riboswitch^c^	VPBB_A1172-VPBB_A1173	100^d^	repressed	0.24
Candidate 12^a^	VPBB_A1578-VPBB_A1579	207^e^	repressed	0.15

^a^sRNAs identified with sRNAPredict2

^b^ Not found in either database

^c^ sRNAs identified with BSRD

^d^ Due to the qRT-PCR size and primer requirements, sRNA expression was not further validated. Only the Srr was confirmed via qRT-PCR since it was not part of one of the existing sRNA databases.

^e^ The approximate genomic location of Srr is 1354895–1355085 bp and Candidate 12 is 1776278–1776403 bp. Due to the lack of strand specific data, the location of the other sRNAs was not determined, although all sRNAs were observed in the RNA-Seq data.

^f^RNA-Seq data is fold change of the *opaR* ^+^ strain gene expression divided by the ∆*opaR1* strain gene expression. Error for the ratios of normalized gene expression levels were conservatively estimated using the standard deviation ratios across the majority of genes with less than 4-fold change. The standard deviation for chromosome 1 is 1.53 and the error for chromosome 2 is 1.59.

## Discussion

The recently published genome sequence of the *V*. *parahaemolyticus* strain BB22OP revealed over 200 genes unique to the BB22OP strain that were not found in the previously sequenced RIMD2210633 strain [16]. Knowing there are differences in the two genomes prompted an examination of the complete *V*. *parahaemolyticus* BB22OP transcriptome as measured through RNA-Seq. Prior microarray data for *V*. *parahaemolyticus* BB22OP was generated using a custom Affymetrix GeneChip designed using the first sequenced *V*. *parahaemolyticus* genome of strain RIMD2210633. The OpaR regulon revealed by these two analyses were found to correlate very highly with one another, with just 18 genes inversely regulated in *opaR* ^+^ versus ∆*opaR1* BB22OP strains. Many of these discordant genes were associated with phosphate transporters suggesting that differences in the medium and or water utilized may have led to these minor discrepancies. The RNA-Seq data was further validated via qRT-PCR for a select subset of 11 transcription factor genes that were highly regulated by OpaR (Table 1). Although absolute levels of regulation observed in the microarray, RNA-Seq and qRT-PCR were not identical, the direction of regulation (activation or repression) was confirmed by all three methods and the degree of regulation was in a similar range (Table 1).

**Fig 4:**
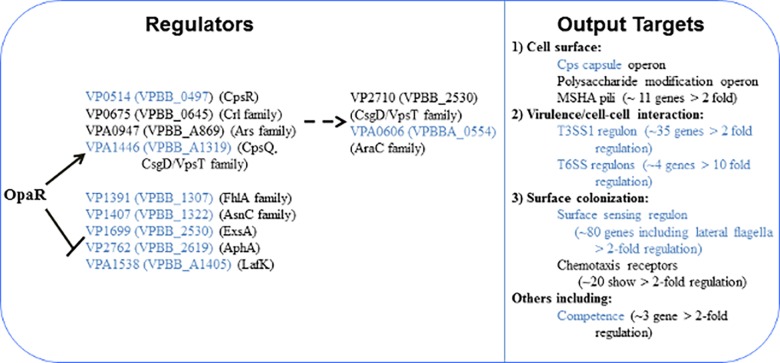
Model of OpaR regulon highlighting downstream transcription factors. Downstream targets of OpaR include 11 transcription factors differentially regulated by OpaR four-fold or more. The genes controlling nine of the transcription factors are directly controlled by OpaR, as indicated by the solid lines. VPA0606 (AraC family protein) and VP2710 (CsgD/VpsT family protein) appear to be indirectly regulated as indicated by the dashed lines. Blue indicates genes about which some experimental data with relation to hierarchical control has been obtained in *V*. *parahaemolyticus*.

The 11 transcription factors downstream of OpaR presumably play an essential role in the regulatory network controlling phenotypic output critical to the survival and virulence of the organism. Transcriptome analyses suggest four major themes with respect to the kinds of genes that are controlled ultimately by OpaR. These include genes pertinent to (1) the cell surface and adhesion, (2) virulence and cell-cell interactions including one type III and two type VI secretion systems, (3) the surface-specific regulon including the lateral flagellar system, chemotaxis and the swarm-specific sRNA, and (4) other functions such as competency (Fig 4).

Of the 11 transcription factors in the OpaR regulon under investigation in this study, the roles of many have been previously examined in *V*. *parahaemolyticus*. The regulators CpsR and CpsQ control capsule production [32,33]. Recent findings show that the AsnC family protein VPBB_1322 and the FhlA family protein VPBB_1307 play roles in the regulation of the type VI secretion system in *V*. *parahaemolyticus* [34]. ExsA, an AraC-family regulator, controls the T3SS1 in *V*. *parahaemolyticus* similar to its role in type-three secretion in other organisms such as *Pseudomonas aeruginosa* [35–37]. The sigma 54-dependent flagellar regulatory protein LafK is known to play a role in swarming motility [35,38–40]. AphA, is a known quorum-sensing regulator that is active primarily at low cell densities. [27]. A mutant with a transposon insertion in the AraC-type regulatory gene VBB_A0554 has a striking biofilm phenotype [41]. The roles of the other three transcription factors are less well defined in *V*. *parahaemolyticus*, but their homologues have been studied in other bacterial species. Crl is a transcriptional activator of the curli formation and fibronectin binding in *Escherichia coli* [42]. The ArsR-type regulator is part of a potential operon encoding a cation efflux transporter; many ArsR family proteins are metalloregulators [43]. VPBB_2530 is a predicted c-di-GMP binding protein homologous to CpsQ and VpsT [32,44].

EMSA analysis confirmed that the genes for nine transcriptional regulators, were direct targets of OpaR while the genes for the remaining two transcription factors appear to be indirectly controlled by OpaR and therefore located further downstream in the regulon (Fig 4). Understanding more about each regulator, what it controls specifically in *V*. *parahaemolyticus*, and how it fits into the cascade of quorum-sensing regulations, can help further understand how *V*. *parahaemolyticus* switches from the nonpathogenic to pathogenic states and is capable of living in several different environments.

The previously designed MQSR box [27] proved to be useful for the theoretical identification of OpaR direct targets when utilized in combination with the RNA-Seq data [27]. OpaR was experimentally determined to bind to a number of sites with low scores in the PATSER program. Since the MQSR box was created using all of the LuxR homologue binding sites, it is perhaps not unexpected that some functional binding sites would have lower scores. In addition to the nine transcription factors experimentally confirmed to be direct targets of OpaR, this work has identified 60 other putative targets direct as well as numerous indirect protein-coding targets of OpaR, and 6 sRNAs predicted in published databases that are differentially expressed by OpaR. In addition, both the microarray and RNA-Seq data have revealed a highly regulated, novel sRNA that is controlled by OpaR. Altogether this study has identified a large number of OpaR-regulated genes. The hierarchy of direct and indirect gene control during quorum sensing is starting to become clear and further study of the roles of the OpaR controlled transcriptional regulators will help to elucidate the downstream quorum sensing cascade critical for ultimately regulating virulence gene expression.

## Supporting Information

S1 Fig Graphical representation of the comparison between the RNA-Seq data and the published microarray data log ratios for chromosome 1 (A) or chromosome 2 (B) of *V*. *parahaemolyticus*. Each point represents a gene reported from the microarray work to have an OpaR fold regulation of four or greater. All genes not along the trend line represent discrepancies in the data. The genes with discrepancies in quadrant II or IV are differentially regulated meaning in one set of data it is repressed and in the second set of data it is activated. For chromosome 1, the genes are mostly DNA uptake related and the differences in chromosome 2 are phosphate transporters (See S3 Table).
(TIF)

S1 Table Primers for qRT-PCR. (DOCX)

S2 Table Primers for amplification of promoter regions. (DOCX)

S3 Table Genes differentially expressed between the microarray and RNA-Seq data. (DOCX)

S4 Table Genes regulated greater than 4-fold in the RNA-Seq data. (DOCX)

S5 Table Additional theoretically predicted putative direct targets of OpaR. (DOCX)
