# Supplementary figures and images for: OpaR Controls a Network of Downstream Transcription Factors in Vibrio parahaemolyticus BB22OP
Source: PLoS One. 2015 Apr 22;10(4):e0121863. doi: 10.1371/journal.pone.0121863 (PMC4406679; doi:10.1371/journal.pone.0121863)

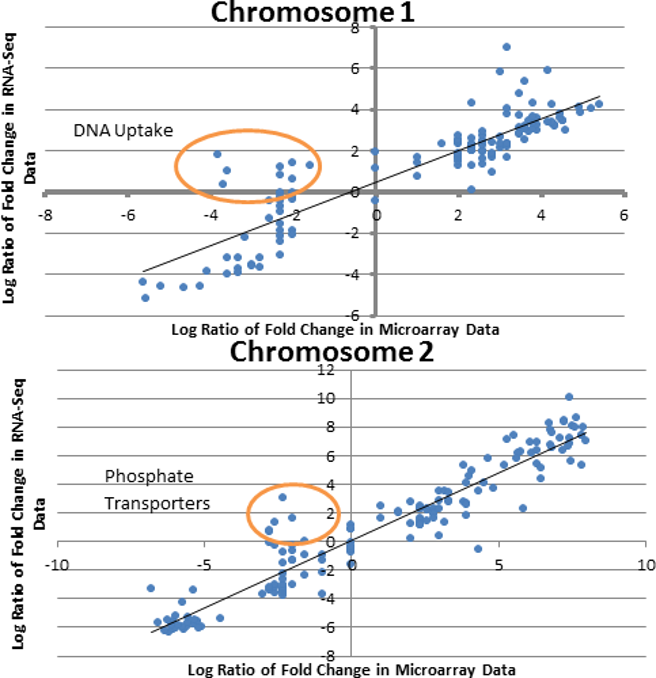

Supplement: S1 Fig — Each point represents a gene reported from the microarray work to have an OpaR fold regulation of four or greater. All genes not along the trend line represent discrepancies in the data. The genes with discrepancies in quadrant II or IV are differentially regulated meaning in one set of data it is repressed and in the second set of data it is activated. For chromosome 1, the genes are mostly DNA uptake related and the differences in chromosome 2 are phosphate transporters (See S3 Table). (TIF) [file pone.0121863.s001.TIF]
