# Supplementary material for: OpaR Controls a Network of Downstream Transcription Factors in Vibrio parahaemolyticus BB22OP
Source: PLoS One. 2015 Apr 22;10(4):e0121863. doi: 10.1371/journal.pone.0121863 (PMC4406679; doi:10.1371/journal.pone.0121863)
Supplement: S1 Table — (DOCX) [file pone.0121863.s002.docx]

**Table S1:** **Primers for qRT-PCR.**

| **Gene** | **Primer** | **Annealing Temp** | **Sequence 5’ to 3’** |
| --- | --- | --- | --- |
| **VPBB_0491 (VP0514)**  ***cpsR*** | Cloning-FWD | 58.5°C | ATGGCTGGGCAGTTTAAGATGG |
|  | Cloning-REV |  | TTACATCGCTTGATCGGAAATCAAG |
|  | qRT-PCR FWD | 60°C | TTGGAGTCGCACTCTGGTCAA |
|  | qRT-PCR REV |  | TGCACGCGACACACCAAGTT |
| **VPBB_0645 (VP0675)**  ***crl* family** | Cloning-FWD | 58.5°C | ATGTCAGAGACGACTCAGGG |
|  | Cloning-REV |  | TCAGGCGAAATCAAATGACTCG |
|  | qRT-PCR FWD | 60°C | CTCAGGGTCCAACACACTTTCG |
|  | qRT-PCR REV |  | GCGGTTCACGCAGGTAAGG |
| **VPBB_2530 (VP2710)**  ***csgD/vpsT* family** | Cloning-FWD | 58.9°C | GTGAGAAAGTCGGCTTACGCAAG |
|  | Cloning-REV |  | TTAAGACATCAAGTTTTGATCGGCCCAAG |
|  | RT-PCR FWD | 62°C | TCGATTAAAAACCTGCCATTGGT |
|  | RT-PCR REV |  | GCGCTTTGGAACGTTGAACA |
| **VPBB_1307 (VP1391)**  ***fhlA* family** | Cloning-FWD | 58.4°C | ATGCGTTCAGCTAACCATAGC |
|  | Cloning-REV |  | TTAACTCGCAGCAGAAATGTCG |
|  | RT-PCR FWD | 60°C | CGCGAAATACATTCACGAAAACTC |
|  | RT-PCR REV |  | AGCCGCACAGTTTTGAACGA |
| **VPBB_1322 (VP1407)**  ***asnC* family** | Cloning-FWD | 61.2°C | ATGATTTCTAATATGACTTTACAGGCTTAGATAAGTTAGATCG |
|  | Cloning-REV |  | CTATCGACTGATCGGCTGATGAAGTAC |
|  | RT-PCR FWD | 62°C | CTGTTAACAAACGGGCGTGAGT |
|  | RT-PCR REV |  | GCGACAGCAGTACGGGACAAA |
| **VPBB_1558 (VP1699)**  ***exsA*** | Cloning-FWD | 58.4°C | ATGGATGTGTCAGGCCAAC |
|  | Cloning-REV |  | TCAATTAGCGATGGCGACTTG |
|  | RT-PCR FWD | 62°C | GGTGAAGTCCTCTATGCCTTGCTATC |
|  | RT-PCR REV |  | TGCTCCATAAAGCGGCGTAA |
| **VPBB_2619 (VP2762)**  ***aphA*** | Cloning-FWD | 56.0°C | TTGTTTACAAGTTTATTGACCATTTGG |
|  | Cloning-REV |  | TTAACCAATCACTTCAAGTTCTGTTAG |
|  | RT-PCR FWD | 64°C | CTTACTACGCAACGCCTTCAACT |
|  | RT-PCR REV |  | CGAAGCGTTAGGCGCTCTA |
| **VPBBA_0554 (VPA606)**  ***araC* family** | Cloning-FWD | 58.4°C | ATGCCGAACATTGAGATCATTCG |
|  | Cloning-REV |  | TTAACCTCTTACTACCTGATTACGAAAGTCTTTTGG |
|  | RT-PCR FWD | 62°C | CCTTTCTCGCCAGAATCCAATT |
|  | RT-PCR REV |  | GCGACTGGCTAAGACCAATAACG |
| **VPBBA_0869**  **(VPA947)**  ***ars* family** | Cloning-FWD | 58.9°C | ATGAGCTATACAGATATGGATGTAGCAGC |
|  | Cloning-REV |  | TTAGTTACAGAAAACGCCATGAAGC |
|  | RT-PCR FWD | 62°C | GCAATGAAAGGCAATGCCAAT |
|  | RT-PCR REV |  | CCTTTCCGGATGTGCCATTAC |
| **VPBBA_1319**  **(VPA1446)**  ***cpsQ*** | Cloning-FWD | 58.3°C | ATGGAACAGTACACGGAAAAGC |
|  | Cloning-REV |  | CTAGAGGTTTCTTTTTGCCCAACTTG |
|  | RT-PCR FWD | 62°C | AGCGTCTTGGCCTCATGTCA |
|  | RT-PCR REV |  | CCTAATCCTCGACAAACCTTTTCC |
| **VPBBA_1405 (VPA1538) *lafK*** | Cloning-FWD | 61.2°C | ATGACGAAAACGAATATTTTGTTGGTTGAGC |
|  | Cloning-REV |  | TTAGGCAGCCGAGCCTAG |
|  | RT-PCR FWD | 62°C | GGGCGAAGCGCACTGTTA |
|  | RT-PCR REV |  | TCCGGTAGCGTAGAGCTCACTAA |
| **VPBBA_0387**  **(VP0404)**  ***rpoD*** | Cloning-FWD | 55°C | TTAGTCATTTTGTGTCCACGCACTG |
|  | Cloning-REV |  | GTGAATAAAGCGATAGAAGCTGCG |
|  | RT-PCR FWD | 62°C | GCCGGCCTGAATTAAATCCT |
|  | RT-PCR REV |  | CACCACCTGTTAGGCCGTTT |
| **VPBBA_2050**  **(VPA1555)**  ***fliA*** | Cloning-FWD | 55°C | TTATTCGTCGAGGAAGCTGCG |
|  | Cloning-REV |  | ATCCGCAGTCACAGATAAAGTTAC |
|  | RT-PCR FWD | 62°C | TTGCATACGCTCTGCCAGTTCT |
|  | RT-PCR REV |  | CCGTATCTCTCGTCAAATGCTACAA |
| **Srr** | Cloning-FWD | 50°C | TGTGCGCTTAGTTGGTTAATC |
|  | Cloning-REV |  | AGTGATTTGTATGTAACTTGATACG |
|  | RT-PCR FWD | 60°C | ATGCATTGCGTCATGGTGAAA |
|  | RT-PCR REV |  | AGGTACGACCACTCCATATAGCTAATGT |
