# Supplementary material for: OpaR Controls a Network of Downstream Transcription Factors in Vibrio parahaemolyticus BB22OP
Source: PLoS One. 2015 Apr 22;10(4):e0121863. doi: 10.1371/journal.pone.0121863 (PMC4406679; doi:10.1371/journal.pone.0121863)
Supplement: S2 Table — (DOCX) [file pone.0121863.s003.docx]

**Table S2:** **Primers for amplification of promoter regions.**

| **Gene** | **Primer Sequence** | **Length of PCR Product (bp)** | **% Gel** |
| --- | --- | --- | --- |
| VPBB_0491 (VP0514)  *cpsR* | Fwd: TTGCATTTTGATTTTGATGGC | 165 | 5% |
|  | Rev: ATCCATCTTAAACTGCCCAG |  |  |
|  | Rev: /FAM/ATCCATCTTAAACTGCCCAG |  |  |
| VPBB_0645 (VP0675)  *crl* family | Fwd: GTTAAGAGTGATATACCTGAGC | 103 | 5% |
|  | Rev: CATATGAATCTCCGTTTCCATATAG |  |  |
|  | Rev: /FAM/CATATGAATCTCCGTTTCCATATAG |  |  |
| VPBB_2619 (VP2762)  *aphA* | Fwd: CAGCAAATAACCAGACATTTACACTAC | 198 | 4% |
|  | Rev: GGAGCAGGTATGATTGGATTTATGAGC |  |  |
|  | Rev:/FAM/GGAGCAGGTATGATTGGATTTATGAGC |  |  |
| VPBB_1307 (VP1391)  *fhlA* family | Fwd: ATAAAGTTAATAACACTGGCATTTTAGTCG | 242 | 5% |
|  | Rev: CGCTGAATTAATGAAGAAAGGT |  |  |
|  | Rev: /FAM/CGCTGAATTAATGAAGAAAGGT |  |  |
| VPBB_1322 (VP1407)  *asnC* family | Fwd: AGGAACAAAGATCACACAAAATG | 206 | 4% |
|  | Rev: CTTGTGAAGTCGCTGAAAAAC |  |  |
|  | Rev: /FAM/CTTGTGAAGTCGCTGAAAAAC |  |  |
| VPBB_1558 (VP1699)  *exsA* | Fwd: GGTTTTGGAAATGTAGTTTCCTAAT | 373 | 4% |
|  | Rev: AATTTCTCTCGTGTGTAAAAGAG |  |  |
|  | Rev: /FAM/AATTTCTCTCGTGTGTAAAAGAG |  |  |
| VPBB_2530 (VP2710) *csgD/vpsT* family | Fwd: CTCAGTTCAACTTTCATATGTAGC | 159 | 6% |
|  | Rev: CACGTGTTCAAATCTCCCAAG |  |  |
|  | Rev: /FAM/CACGTGTTCAAATCTCCCAAG |  |  |
| VPBB_A0554 (VPA0606)  *araC family* | Fwd: CTGTAAGTGAAGAATAATACCGAATG | 123 | 6% |
|  | Rev: AAGTGGTTAAAGCGAATGATC |  |  |
|  | Rev: /FAM/AAGTGGTTAAAGCGAATGATC |  |  |
| VPBB_A0869 (VPA0947)  *arsR family* | Fwd: GAGAACGCCATCCTTTG | 164 | 5% |
|  | Rev: CTGTATAGCTCATGACTCACC |  |  |
|  | Rev: /FAM/CTGTATAGCTCATGACTCACC |  |  |
| VPBB_A1319 (VPA1446)  *cpsQ* | Fwd: ATAGCCTTAGTTAAAAGTGTTTCTTT | 174 | 5% |
|  | Rev: CCATAATGTTTCTCATACTTTCATTAAC |  |  |
|  | Rev: /FAM/CCATAATGTTTCTCATACTTTCATTAAC |  |  |
| VPBB_A1405 (VPA1538)  *lafK* | Fwd: AACTTCGAATTATGTGACTTTAGTTC | 315 | 4% |
|  | Rev: TGGCATAGCTAATCATGTTAATTAC |  |  |
|  | Rev: /FAM/TGGCATAGCTAATCATGTTAATTAC |  |  |
| *fliA* | Fwd: AAGTGCTGGCGTTTGTGTTC | 276 | 4% |
|  | Rev: TTAACTTCATACTGCGAGGTATC |  |  |
|  | Rev: /FAM/ TTAACTTCATACTGCGAGGTATC |  |  |
