# Supplementary material for: OpaR Controls a Network of Downstream Transcription Factors in Vibrio parahaemolyticus BB22OP
Source: PLoS One. 2015 Apr 22;10(4):e0121863. doi: 10.1371/journal.pone.0121863 (PMC4406679; doi:10.1371/journal.pone.0121863)
Supplement: S3 Table — (DOCX) [file pone.0121863.s004.docx]

**Table S3:** **Genes differentially expressed between the microarray and RNA-Seq data**

| **BB22OP ID** | **RIMD**  **2210633 ID** | **Annotation** | **Microarray Fold Change^a^** | ***opaR*^+^/**  **Δ*opaR1***  **RNA-Seq^b^** |
| --- | --- | --- | --- | --- |
| **Chromosome 1** | | | | |
| VPBB_0158 | VP0167 | hypothetical | 0.20 | 0.99 |
| VPBB_0556 | VP0583 | malate synthase A | 0.25 | 2.69 |
| VPBB_0557 | VP0584 | isocitrate lyase | 0.20 | 1.74 |
| VPBB_0880 | VP0922 | competence related protein | 0.08 | 1.31 |
| VPBB_2153 | VP2338 | chitinase (SSR^c^) | 5.00 | 1.06 |
| VPBB_2718 | VP2868 | hypothetical protein | 0.07 | 0.63 |
| VPBB_2719 | VP2869 | sodium solute symporter | 0.08 | 2.03 |
| VPBB_2725 | VP2876 | conserved hypothetical protein (SSR) | 0.25 | 1.52 |
| VPBB_2726 | VP2877 | DNA polymerase III, epsilon subunit (SSR) | 0.33 | 2.41 |
| VPBB_2727 | VP2878 | acetyl-CoA synthase (SSR) | 0.25 | 2.34 |
| **Chromosome 2** | | | | |
| VPBBA_1424 | VPA1558 | FliU, new potential lateral product (SSR; last gene in a 9-gene operon) | 20.00 | 0.67 |
| VPBBA_0324 | VPA0352 | permease of ABC transporter | 0.33 | 1.02 |
| VPBBA_1333 | VPA1460 | phosphate ABC transporter, permease protein | 0.25 | 3.41 |
| VPBBA_1334 | VPA1461 | phosphate ABC transporter, periplasmic phosphate-binding | 0.20 | 8.40 |
| VPBBA_1331 | VPA1458 | phosphate ABC transporter, ATP-binding protein | 0.17 | 2.50 |
| VPBBA_0326 | VPA0354 | periplasmic solute-binding protein | 0.14 | 1.64 |
| VPBBA_0528 | VPA0579 | alkaline phosphatase | 0.14 | 0.92 |
| VPBBA_1332 | VPA1459 | phosphate ABC transporter, permease protein | 0.14 | 1.70 |

| ^a^Tests for differential expression were conducted using an ANOVA model in R with an FDR = 0.03 when expression in strain LM5674 (∆*opaR1*) was compared to LM5312 (*opaR^+^*). Inclusion in this list was determined by change in expression of 4-fold or greater. [[13](#_ENREF_13)]  ^b^ RNA-Seq data is fold change of the *opaR*^+^ strain gene expression divided by the ∆*opaR1* strain gene expression. Error for the ratios of normalized gene expression levels were conservatively estimated using the standard deviation ratios across the majority of genes with less than 4-fold change. The standard deviation for chromosome 1 is 1.53 and the error for chromosome 2 is 1.59.  ^c^SSR= surface sensing regulon |
| --- |
