# Supplementary material for: OpaR Controls a Network of Downstream Transcription Factors in Vibrio parahaemolyticus BB22OP
Source: PLoS One. 2015 Apr 22;10(4):e0121863. doi: 10.1371/journal.pone.0121863 (PMC4406679; doi:10.1371/journal.pone.0121863)
Supplement: S4 Table — (DOCX) [file pone.0121863.s005.docx]

**Table S4: Genes regulated greater than 4-fold in the RNA-Seq data.**

| **BB22OP ID ^a^** | **RIMD**  **2210633**  **ID ^b^** | ***opaR*^+^/**  **Δ*opaR1***  **RNA-Seq^c^** | | **Annotation** |
| --- | --- | --- | --- | --- |
| **Chromosome 1** | | | | |
| VPBB_0006 | VP0006 | 6.61 | | ABC-type polar amino acid transport system, ATPase component |
| VPBB_0008 | VP0008 | 19.58 | | amino acid ABC transporter, periplasmic amino acid-binding portion |
| VPBB_0048 | VP0053 | 59.73 | | hypothetical protein |
| VPBB_0050 | VP0055 | 5.48 | | DNA-directed RNA polymerase specialized sigma subunit |
| VPBB_0051 | VP0056 | 5.03 | | chromosome segregation ATPase |
| VPBB_0052 | VP0057 | 5.79 | | serine/threonine protein kinase |
| VPBB_0107 | VP0117 | 7.74 | | GGDEF and EAL family protein |
| VPBB_0384 | VP0377 | 0.25 | | putative CFA |
| **VPBB_0491** | **VP0514** | **0.17** | | **capsule transcriptional regulator CpsR** |
| VPBB_0535 | VP0562 | 6.18 | | hypothetical protein |
| VPBB_0596 | VP0625 | 0.04 | | hypothetical protein |
| VPBB_0597 | VP0626 | 0.07 | | hypothetical protein |
| VPBB_0598 | VP0627 | 0.11 | | hypothetical protein |
| VPBB_0620 | VP0649 | 4.52 | | pypothetical protein |
| VPBB_0623 | VP0652 | 19.04 | | proton glutamate symport protein |
| VPBB_0670 | VP0699 | 9.96 | | GGDEF family protein |
| VPBB_0732 | VP0766 | 16.27 | | hypothetical protein, specific for Vibrio |
| VPBB_0755 | VP0790 | 4.45 | | polar flagellin protein FlaD |
| VPBB_0860 | VP0901 | 19.98 | | hypothetical protein |
| VPBB_0905 | VP0948 | 4.38 | | D-Tyr-tRNAtyr deacylase |
| VPBB_0907 | VP0950 | 0.19 | | lipoprotein-related protein |
| VPBB_0953 | VP1002 | 7.84 | | hypothetical protein |
| VPBB_1018 | VP1088 | 4.95 | | methyl-accepting chemotaxis protein I (serine chemoreceptor protein) |
| VPBB_1164 | VP1242 | 0.12 | | hypothetical protein |
| VPBB_1177 | VP1254 | 4.81 | | hypothetical protein |
| VPBB_1211 | VP1286 | 0.24 | | putative exporter of the RND superfamily |
| VPBB_1212 | VP1287 | 0.21 | | outer membrane lipoprotein-sorting protein |
| VPBB_1213 | VP1288 | 0.25 | | hypothetical protein |
| VPBB_1242 | VP1318 | 0.08 | | hypothetical protein |
| VPBB_1243 | VP1319 | 0.09 | | polysaccharide pyruvyl transferase |
| VPBB_1244 | VP1320 | 0.07 | | 2-C-methyl-D-erythritol 4-phosphate cytidylyltransferase |
| VPBB_1245 | VP1321 | 0.08 | | dTDP-glucose 4,6-dehydratase |
| VPBB_1246 | VP1322 | 0.11 | | lipopolysaccharide cholinephosphotransferase LicD1 |
| VPBB_1256 | VP1332 | 4.64 | | ABC transporter, periplasmic spermidine putrescine-binding protein PotD |
| VPBB_1258 | VP1335 | 6.45 | | 1-pyrroline-4-hydroxy-2-carboxylate deaminase |
| VPBB_1302 | VP1385 | 5.31 | | cell wall endopeptidase, family M23 M37 |
| VPBB_1303 | VP1387 | 10.98 | | hypothetical protein |
| VPBB_1304 | VP1388 | 4.23 | | hypothetical protein |
| **VPBB_1307** | **VP1391** | **16.21** | | **sigma-54 dependent transcriptional regulator, FhlA family** |
| VPBB_1308 | VP1392 | 14.23 | | ClpB protein |
| VPBB_1309 | VP1393 | 26.72 | | typeVI secretion Hcp protein, biofilm development |
| VPBB_1310 | VP1394 | 19.28 | | VgrG protein |
| VPBB_1311 | VP1395 | 10.99 | | hypothetical protein |
| VPBB_1312 | VP1396 | 8.12 | | hypothetical protein |
| VPBB_1313 | VP1398 | 8.25 | | hypothetical protein |
| VPBB_1314 | VP1399 | 10.10 | | hypothetical protein |
| VPBB_1315 | VP1400 | 17.45 | | hypothetical protein |
| VPBB_1316 | VP1401 | 13.50 | | uncharacterized protein ImpA |
| VPBB_1317 | VP1402 | 12.19 | | uncharacterized protein ImpB |
| VPBB_1318 | VP1403 | 10.47 | | uncharacterized protein ImpC |
| VPBB_1319 | VP1404 | 10.87 | | hypothetical protein |
| VPBB_1320 | VP1405 | 11.93 | | protein ImpG |
| VPBB_1321 | VP1406 | 7.77 | | uncharacterized protein ImpH |
| VPBB_1323 | VP1408 | 9.32 | | IcmF-related protein |
| VPBB_1324 | VP1409 | 7.22 | | uncharacterized protein ImpA |
| VPBB_1325 | VP1410 | 10.89 | | hypothetical protein |
| VPBB_1326 | VP1411 | 9.75 | | uncharacterized protein ImpI |
| VPBB_1327 | VP1412 | 10.45 | | type VI secretion lipoprotein |
| VPBB_1328 | VP1413 | 9.35 | | uncharacterized protein ImpJ |
| VPBB_1329 | VP1414 | 8.47 | | outer membrane protein ImpK |
| VPBB_1330 | VP1415 | 4.56 | | hypothetical protein |
| VPBB_1331 |  | 4.48 | | hypothetical protein |
| VPBB_1332 |  | 4.58 | | hypothetical protein |
| VPBB_1335 |  | 5.36 | | hypothetical protein |
| VPBB_1336 | VP1422 | 6.51 | | SM-20-related protein |
| VPBB_1345 | VP1434 | 0.11 | | fimbrial protein pilin |
| VPBB_1415 | VP1510 | 4.52 | | iron-sulfur cluster-binding protein |
| VPBB_1418 | VP1513 | 4.49 | | formate dehydrogenase-O, major subunit |
| VPBB_1419 | VP1514 | 5.68 | | formate dehydrogenase-O, iron-sulfur subunit |
| VPBB_1420 | VP1515 | 4.40 | | formate dehydrogenase -O, gamma subunit |
| VPBB_1421 | VP1516 | 4.24 | | hypothetical protein |
| VPBB_1422 | VP1517 | 0.03 | | hypothetical protein |
| VPBB_1423 | VP1521 | 0.05 | | hypothetical protein |
| VPBB_1424 | VP1522 | 0.06 | | hypothetical protein |
| VPBB_1425 |  | 0.06 | | hypothetical protein |
| VPBB_1426 | VP1521 | 0.13 | | hypothetical protein |
| VPBB_1494 | VP1634 | 0.12 | | agglutination protein |
| VPBB_1495 | VP1635 | 0.12 | | putative outer membrane protein |
| VPBB_1518 | VP1658 | 5.09 | | type III secretion chaperone protein for YopD (SycD) |
| VPBB_1526 | VP1666 | 5.63 | | type III secretion outermembrane negative regulator of secretion (TyeA) |
| VPBB_1527 | VP1667 | 5.07 | | type III secretion outermembrane contact sensing protein (YopN,Yop4b,LcrE) |
| VPBB_1528 | VP1668 | 6.31 | | type III secretion ATPase |
| VPBB_1541 | VP1682 | 7.05 | | hypothetical protein |
| VPBB_1546 | VP1688 | 4.66 | | type III secretion cytoplasmic protein (YscL) |
| VPBB_1548 | VP1690 | 4.06 | | type III secretion bridge between inner and outermembrane lipoprotein (YscJ,HrcJ,EscJ, PscJ) |
| VPBB_1549 | VP1691 | 4.86 | | type III secretion cytoplasmic protein (YscI) |
| VPBB_1554 | VP1695 | 4.25 | | type III secretion inner membrane protein (YscD, flagellar export protein-like protein) |
| VPBB_1555 | VP1696 | 4.22 | | type III secretion outermembrane pore forming protein (YscC,MxiD,HrcC, InvG) |
| VPBB_1560 | VP1701 | 12.70 | | type III secretion regulator ExsC |
| VPBB_1561 | VP1702 | 13.45 | | type III secretion regulator ExsE |
| VPBB_1628 | VP1770 | 0.25 | | manganese-dependent inorganic pyrophosphatase |
| VPBB_1633 | VP1775 | 4.73 | | gamma-glutamyl-putrescine oxidase |
| VPBB_1636 | VP1779 | 57.78 | | gamma-glutamyl-GABA hydrolase |
| VPBB_1637 | VP1781 | 10.31 | | gamma-glutamyl-putrescine synthetase |
| VPBB_1646 | VP1864 | 0.19 | | glyoxalase family protein |
| VPBB_1647 |  | 0.10 | | hypothetical protein |
| VPBB_1648 |  | 0.24 | | hypothetical protein |
| VPBB_1649 |  | 0.18 | | hypothetical protein |
| VPBB_1650 |  | 0.14 | | hypothetical protein |
| VPBB_1652 |  | 0.10 | | hypothetical protein |
| VPBB_1655 |  | 0.25 | | hypothetical protein |
| VPBB_1669 |  | 0.15 | | hypothetical protein |
| VPBB_1670 |  | 0.24 | | putative acetyltransferase |
| VPBB_1681 |  | 0.21 | | hypothetical protein |
| VPBB_1693 |  | 0.18 | | hypothetical protein |
| VPBB_1694 |  | 0.21 | | L-2-haloalkanoic acid dehalogenase |
| VPBB_1696 |  | 0.16 | | hypothetical protein |
| VPBB_1697 |  | 0.20 | | hypothetical protein |
| VPBB_1704 |  | 0.22 | | hypothetical protein |
| VPBB_1711 | VP1815 | 0.10 | | hypothetical protein |
| VPBB_1730 | VP1879 | 5.15 | | serine transporter |
| VPBB_1732 | VP1881 | 5.97 | | EAL family protein |
| VPBB_1745 | VP1904 | 9.12 | | methyl-accepting chemotaxis protein |
| VPBB_1796 | VP1955 | 0.23 | | hypothetical protein |
| VPBB_1805 | VP1966 | 6.37 | | proton glutamate symport protein |
| VPBB_1817 | VP1979 | 0.23 | | EAL family protein |
| VPBB_1848 | VP2011 | 4.09 | | tetrathionate reductase subunit B |
| VPBB_1851 | VP2015 | 128.99 | | cytochrome c4 |
| VPBB_1852 | VP2016 | 40.40 | | cytochrome c family protein |
| VPBB_1857 | VP2021 | 0.21 | | hypothetical protein |
| VPBB_1858 | VP2022 | 0.17 | | putative glycosyl transferase family protein |
| VPBB_1859 | VP2023 | 0.19 | | dTDP-glucose 4,6-dehydratase |
| VPBB_1954 | VP2119 | 4.12 | | putative queD like 2 |
| VPBB_1979 | VP2159 | 6.01 | | methyl-accepting chemotaxis protein |
| VPBB_2025 | VP2207 | 0.23 | | putative CG2 omega domain protein |
| VPBB_2029 | VP2211 | 5.33 | | hypothetical protein |
| VPBB_2053 | VP2235 | 6.77 | | polar flagellar biosynthesis protein FlhA |
| VPBB_2072 | VP2254 | 4.04 | | polar flagellar regulatory protein FlaJ |
| VPBB_2073 | VP2255 | 4.81 | | polar flagellar protein FlaI |
| VPBB_2074 | VP2256 | 4.13 | | polar flagellar hook-associated protein FliD |
| VPBB_2076 | VP2258 | 4.93 | | polar flagellin protein FlaA |
| VPBB_2077 | VP2259 | 4.44 | | polar flagellin protein FlaB |
| VPBB_2078 | VP2261 | 5.46 | | flagellin protein FlaF |
| VPBB_2144 | VP2329 | 13.13 | | multidrug efflux pump component MtrF |
| VPBB_2269 | VP2443 | 0.16 | | hypothetical protein |
| VPBB_2301 |  | 5.66 | | hypothetical protein |
| VPBB_2338 | VP2516 | 0.06 | | quorum-sensing regulator OpaR |
| VPBB_2451 | VP2631 | 8.87 | | HD-GYP family protein |
| VPBB_2456 | VP2636 | 0.16 | | PTS system, cellobiose-specific IIC component |
| VPBB_2457 | VP2637 | 0.23 | | PTS system, cellobiose-specific IIB component |
| VPBB_2458 | VP2638 | 0.21 | | deacetylase DA1 |
| **VPBB_2619** | **VP2762** | **5.09** | | **transcriptional regulator, PadR family, AphA** |
| VPBB_2661 | VP2811 | 4.78 | | polar Na+-driven flagellar motor protein MotX |
| VPBB_2677 | VP2827 | 5.91 | | methyl-accepting chemotaxis protein |
| VPBB_2736 | VP2888 | 4.44 | | GGDEF family protein |
| VPBB_2755 | VP2917 | 0.04 | | hypothetical protein |
| VPBB_2756 | VP2918 | 0.04 | | hypothetical protein |
| VPBB_2811 | VP2974 | 5.43 | | lysophospholipase L2 |
| VPBB_2816 | VP2979 | 0.24 | | GGDEF & EAL family protein with FIST domain |
| VPBB_2845 | VP3014 | 14.30 | | putative signal peptide protein |
| VPBB_2846 | VP3015 | 7.32 | | hypothetical protein |
| VPBB_2893 |  | 4.28 | | hypothetical protein |
| VPBB_2894 |  | 4.53 | | hypothetical protein |
| **Chromosome 2** | | | | |
| VPBB_A0045 | VPA0052 | | 0.17 | fucose 4-O-acetylase |
| VPBB_A0095 | VPA0104 | | 0.23 | lactoylglutathione lyase |
| VPBB_A0112 | VPA0121 | | 0.16 | hypothetical protein |
| VPBB_A0189 | VPA0207 | | 5.55 | peptide methionine sulfoxide reductase MsrA |
| VPBB_A0227 | VPA0248 | | 0.19 | outer membrane protein A precursor |
| VPBB_A0232 | VPA0253 | | 6.84 | putative transport protein |
| VPBB_A0239 | VPA0260 | | 7.1 | FlgO lateral flagellar protein |
| VPBB_A0240 | VPA0261 | | 39.29 | FlgN lateral flagellar chaperone protein |
| VPBB_A0241 | VPA0262 | | 55 | FlgM lateral flagellar anti-sigma factor |
| VPBB_A0243 | VPA0264 | | 132.64 | FlgB lateral flagellar proximal rod protein |
| VPBB_A0244 | VPA0265 | | 274.62 | FlgC lateral flagellar proximal rod protein |
| VPBB_A0245 | VPA0266 | | 254.11 | FlgD lateral flagellar hook assembly protein |
| VPBB_A0246 | VPA0267 | | 342.77 | FlgE lateral flagellar hook protein |
| VPBB_A0247 | VPA0268 | | 145.42 | FlgF lateral flagellar proximal rod protein |
| VPBB_A0248 | VPA0269 | | 195.91 | FlgG lateral flagellar distal rod protein |
| VPBB_A0249 | VPA0270 | | 140.05 | FlgH lateral flagellar L-ring protein |
| VPBB_A0250 | VPA0271 | | 72.8 | FlgI lateral flagellar P-ring protein |
| VPBB_A0251 | VPA0272 | | 77.66 | FlgJ lateral flagellar peptidoglycan hydrolase |
| VPBB_A0252 | VPA0273 | | 153.28 | FlgK lateral flagellar hook-associated protein 1 |
| VPBB_A0253 | VPA0274 | | 94.13 | FlgL lateral flagellar hook-associated protein 3 |
| VPBB_A0254 | VPA0275 | | 12.86 | FlgU lateral flagellar protein |
| VPBB_A0257 | VPA0277 | | 0.22 | NhaP-type antiporter |
| VPBB_A0379 | VPA0409 | | 0.17 | plasma membrane protein involved in salt tolerance |
| VPBB_A0412 | VPA0457 | | 0.18 | hypothetical protein |
| VPBB_A0413 |  | | 0.11 | hypothetical protein |
| VPBB_A0414 | VPA0458 | | 0.11 | hypothetical protein |
| VPBB_A0415 | VPA0459 | | 5.02 | surface-induced secreted collagenase |
| VPBB_A0440 | VPA0485 | | 4.55 | NAD-dependent formate dehydrogenase alpha subunit |
| VPBB_A0446 | VPA0491 | | 5.53 | methyl-accepting chemotaxis protein |
| VPBB_A0456 | VPA0502 | | 5.41 | putative oxidoreductase linked to yggC |
| VPBB_A0464 | VPA0511 | | 23.68 | methyl-accepting chemotaxis protein |
| VPBB_A0470 | VPA0518 | | 0.12 | GGDEF & EAL family protein |
| VPBB_A0514 | VPA0567 | | 0.1 | sigma cross-reacting protein 27A |
| VPBB_A0515 | VPA0568 | | 0.12 | hypothetical protein |
| VPBB_A0537 | VPA0588 | | 0.22 | hypothetical protein |
| **VPBB_A0554** | **VPA0606** | | **0.16** | **biofilm regulatory protein AraC family** |
| VPBB_A0559 | VPA0612 | | 11.3 | methyl-accepting chemotaxis protein |
| VPBB_A0688 | VPA0747 | | 16.87 | MSHA pilin protein MshA |
| VPBB_A0720 | VPA0780 | | 0.2 | hypothetical protein |
| VPBB_A0725 | VPA0783 | | 0.24 | hypothetical protein |
| VPBB_A0729 | VPA0788 | | 0.24 | Phospholipase A1 precursor |
| VPBB_A0866 | VPA0944 | | 0.22 | Acriflavin resistance protein |
| VPBB_A0867 | VPA0945 | | 0.2 | putative transporter component |
| VPBB_A0868 | VPA0946 | | 0.08 | putative transmembrane protein |
| **VPBB_A0869** | **VPA0947** | | **0.12** | **transcriptional regulator ArsR family** |
| VPBB_A0910 | VPA1000 | | 4.99 | methyl-accepting chemotaxis protein I |
| VPBB_A0931 | VPA1024 | | 0.02 | transhydrogenase beta subunit |
| VPBB_A0932 | VPA1026 | | 0.02 | VgrG protein |
| VPBB_A0933 | VPA1027 | | 0.01 | putative cytoplasmic protein USSDB7A |
| VPBB_A0934 | VPA1028 | | 0.02 | ClpA/B-type chaperone |
| VPBB_A0935 | VPA1029 | | 0.03 | hypothetical protein |
| VPBB_A0936 | VPA1030 | | 0.02 | protein ImpG/VasA |
| VPBB_A0937 | VPA1031 | | 0.02 | uncharacterized protein ImpF |
| VPBB_A0938 | VPA1032 | | 0.01 | protein of avirulence locus ImpE |
| VPBB_A0939 | VPA1033 | | 0.02 | uncharacterized protein ImpD |
| VPBB_A0940 | VPA1034 | | 0.01 | uncharacterized protein ImpC |
| VPBB_A0941 | VPA1035 | | 0.02 | uncharacterized protein ImpB |
| VPBB_A0942 | VPA1036 | | 0.02 | uncharacterized protein ImpA |
| VPBB_A0943 | VPA1037 | | 0.02 | putative phosphoprotein phosphatase |
| VPBB_A0944 | VPA1038 | | 0.02 | protein phosphatase ImpM |
| VPBB_A0945 | VPA1039 | | 0.02 | IcmF-related protein |
| VPBB_A0946 | VPA1040 | | 0.02 | type VI secretion system family protein |
| VPBB_A0947 | VPA1041 | | 0.02 | hypothetical protein |
| VPBB_A0948 | VPA1042 | | 0.02 | type VI secretion protein |
| VPBB_A0949 | VPA1043 | | 0.01 | hypothetical protein |
| VPBB_A0950 | VPA1044 | | 0.02 | protein kinase |
| VPBB_A0953 | VPA1047 | | 0.23 | hypothetical protein |
| VPBB_A0985 | VPA1081 | | 17.09 | hypothetical protein |
| VPBB_A0994 | VPA1091 | | 5.93 | hypothetical protein |
| VPBB_A1088 | VPA1189 | | 4.81 | methyl-accepting chemotaxis protein I (serine chemoreceptor protein) |
| VPBB_A1102 | VPA1202 | | 0.09 | polyhydroxyalkanoic acid synthase |
| VPBB_A1103 | VPA1203 | | 0.1 | hypothetical protein |
| VPBB_A1104 | VPA1204 | | 0.05 | 3-ketoacyl-CoA thiolase |
| VPBB_A1105 | VPA1205 | | 0.01 | acetoacetyl-CoA reductase |
| VPBB_A1175 | VPA1294 | | 36.15 | surface-induced SPOR-domain containing protein |
| VPBB_A1276 | VPA1403 | | 0.07 | capsular polysaccharide synthesis enzyme CpsA sugar transferase |
| VPBB_A1277 | VPA1404 | | 0.08 | capsular polysaccharide synthesis enzyme CpsB |
| VPBB_A1278 | VPA1405 | | 0.11 | capsular polysaccharide synthesis enzyme CpsC polysaccharide export |
| VPBB_A1279 | VPA1406 | | 0.12 | capsular polysaccharide synthesis enzyme CpsD exopolysaccharide synthesis |
| VPBB_A1280 | VPA1407 | | 0.11 | capsular polysaccharide synthesis enzyme CpsE |
| VPBB_A1281 | VPA1408 | | 0.08 | glycosyltransferase CpsF |
| VPBB_A1282 | VPA1409 | | 0.09 | capsular polysaccharide synthesis enzyme CpsG Lipid A core - O-antigen ligase |
| VPBB_A1283 | VPA1410 | | 0.1 | capsular polysaccharide synthesis enzyme CpsH |
| VPBB_A1284 | VPA1411 | | 0.08 | putative glycosyltransferase CpsI |
| VPBB_A1285 | VPA1412 | | 0.1 | capsular polysaccharide synthesis enzyme CpsJ membrane protein export of O-antigen and teichoic acid |
| VPBB_A1286 | VPA1413 | | 0.15 | hypothetical protein CpsK |
| VPBB_A1307 | VPA1434 | | 4.94 | ABC transporter ATP-binding protein |
| VPBB_A1308 | VPA1435 | | 4.76 | ferrichrome-iron receptor |
| VPBB_A1310 | VPA1437 | | 4.05 | ferrichrome-binding periplasmic protein precursor |
| VPBB_A1316 | VPA1443 | | 0.17 | membrane-fusion protein MfpC |
| VPBB_A1317 | VPA1444 | | 0.16 | putative transport protein MfpB |
| VPBB_A1318 | VPA1445 | | 0.21 | cell surface and biofilm determinant MfpA |
| **VPBB_A1319** | **VPA1446** | | **0.12** | **transcriptional regulator CpsQ** |
| VPBB_A1320 | VPA1447 | | 0.16 | transcriptional regulator CpsS |
| VPBB_A1322 | VPA1449 | | 7.44 | methyl-accepting chemotaxis protein I (chemoreceptor protein) |
| VPBB_A1334 | VPA1461 | | 8.4 | phosphate ABC transporter periplasmic phosphate-binding protein PstS |
| VPBB_A1335 | VPA1462 | | 10.9 | methyl-accepting chemotaxis protein I (chemoreceptor protein) |
| VPBB_A1362 | VPA1492 | | 6.41 | methyl-accepting chemotaxis protein I (serine chemoreceptor protein) |
| VPBB_A1369 | VPA1499 | | 0.15 | L-lactate dehydrogenase |
| VPBB_A1379 | VPA1511 | | 6.91 | GGDEF and EAL family protein ScrC |
| VPBB_A1380 | VPA1512 | | 6.93 | extracellular solute binding protein ScrB |
| VPBB_A1381 | VPA1513 | | 11.15 | aminotransferase ScrA |
| VPBB_A1384 | VPA1516 | | 4.67 | C4-type zinc finger protein |
| VPBB_A1385 | VPA1518 | | 4.34 | hypothetical protein |
| VPBB_A1386 | VPA1519 | | 8.31 | hypothetical protein |
| VPBB_A1399 | VPA1532 | | 41.44 | FliJ lateral assembly flagellar protein |
| VPBB_A1400 | VPA1533 | | 43.79 | FliI lateral flagellum-specific ATP synthase |
| VPBB_A1401 | VPA1534 | | 72.76 | FliH lateral flagellar assembly protein |
| VPBB_A1402 | VPA1535 | | 125.9 | FliG lateral flagellar motor switch protein |
| VPBB_A1403 | VPA1536 | | 99.91 | FliF lateral flagellar MS-ring protein |
| VPBB_A1404 | VPA1537 | | 216.61 | FliE lateral flagellar hook-basal body MS-ring-rod junction protein |
| **VPBB_A1405** | **VPA1538** | | **170.17** | **LafK sigma 54-dependent lateral flagellar regulatory protein** |
| VPBB_A1406 | VPA1539 | | 306.68 | MotY lateral flagellar H+-driven motor protein |
| VPBB_A1407 | VPA1540 | | 114.21 | FliM lateral flagellar motor protein |
| VPBB_A1408 | VPA1541 | | 81 | FliN lateral fgellar motor protein |
| VPBB_A1409 | VPA1542 | | 56.28 | FliP lateral flagellar protein |
| VPBB_A1410 | VPA1543 | | 124.23 | FliQ lateral flagellar protein |
| VPBB_A1411 | VPA1544 | | 31.38 | FliR lateral flagellar protein |
| VPBB_A1412 | VPA1545 | | 16.57 | FlhB lateral flagellar protein |
| VPBB_A1413 | VPA1546 | | 6.15 | FlhA lateral flagellar protein |
| VPBB_A1415 | VPA1548 | | 1056.05 | LafA lateral flagellin protein |
| VPBB_A1416 | VPA1550 | | 321.76 | FliD lateral flagellar distal cap protein |
| VPBB_A1417 | VPA1551 | | 257.23 | FliS lateral flagellar chaperone protein |
| VPBB_A1418 | VPA1552 | | 401.56 | FliT lateral flagellar chaperone protein |
| VPBB_A1419 | VPA1553 | | 170.31 | FliK lateral flagellar hook-length control protein |
| VPBB_A1420 | VPA1554 | | 169.13 | FliL lateral flagellar protein |
| VPBB_A1421 | VPA1555 | | 39.63 | FliA lateral flagellar RNA polymerase sigma factor |
| VPBB_A1422 | VPA1556 | | 108.25 | MotA lateral flagellar H+-driven motor rotation protein |
| VPBB_A1423 | VPA1557 | | 47.71 | MotB lateral flagellar H+-driven motor rotation protein |
| VPBB_A1434 | VPA1570 | | 11.37 | hypothetical protein |
| VPBB_A1461 | VPA1598 | | 20.32 | surface-induced N-acetyl glucosamine-Chitin binding protein GpbA |
| VPBB_A1473 |  | | 0.1 | putative lipase |
| VPBB_A1474 | VPA1610 | | 0.21 | hypothetical protein |
| VPBB_A1498 | VPA1634 | | 4.45 | antiporter PotE |
| VPBB_A1499 | VPA1635 | | 18.23 | ornithine decarboxylase |
| VPBB_A1501 | VPA1637 | | 4.84 | TPR repeat protein SEL1 subfamily |
| VPBB_A1512 | VPA1649 | | 82.29 | surface-induced metalloendoprotease LytM (M23 family) |
| VPBB_A1513 |  | | 66.78 | hypothetical protein |

^a^Bold indicates genes chosen for further study.

**^b^**Empty RIMD cells indicate genes unique to BB22OP, therefore no RIMD2210633 ID is available.

^c^  RNA-Seq data is fold change of the *opaR^+^* strain gene expression divided by the ∆*opaR1* strain gene expression. Error for the ratios of normalized gene expression levels were conservatively estimated using the standard deviation ratios across the majority of genes with less than 4-fold change. Error for chromosome 1 is 1.53 and error for chromosome 2 is 1.59.
