# Supplementary material for: OpaR Controls a Network of Downstream Transcription Factors in Vibrio parahaemolyticus BB22OP
Source: PLoS One. 2015 Apr 22;10(4):e0121863. doi: 10.1371/journal.pone.0121863 (PMC4406679; doi:10.1371/journal.pone.0121863)
Supplement: S5 Table — (DOCX) [file pone.0121863.s006.docx]

**Table S5**: **Additional theoretically predicted direct targets of OpaR.**

| **BB22OP**  **ID** | **RIMD**  **2210633**  **ID** | **PATSER Score^a^** | **OpaR-binding site 5’ end** | **OpaR-binding site 3’ end** | **Protein Function** | ***opaR*^+^/**  **Δ*opaR1***  **RNA-Seq^b,c^** |
| --- | --- | --- | --- | --- | --- | --- |
|  |  |  |  |  |  |  |
| VPBB_0008 | VP0008 | 7.59 | -53 | -34 | amino acid ABC transporter, periplasmic amino acid-binding portion | 19.58 |
| VPBB_0048 | VP0053 | 6.63 | -241 | -222 | hypothetical protein | 59.72 |
| VPBB_0052 | VP0057 | 3.32 | -52 | -33 | serine/threonine protein kinase | 5.8 |
| VPBB_0107 | VP0117 | 11.77 | -55 | -36 | GGDEF and EAL family protein | 7.74 |
| VPBB_0623 | VP0652 | 8.24 | -131 | -112 | proton glutamate symport protein | 19.04 |
| VPBB_0670 | VP0699 | 7.73 | -64 | -45 | GGDEF family protein | 10 |
| VPBB_0732 | VP0766 | 5.78 | -353 | -334 | hypothetical protein, specific for Vibrio | 16.27 |
| VPBB_0860 | VP0901 | 5.73 | -29 | -10 | hypothetical protein | 20 |
| VPBB_0907 | VP0950 | 4.07 | -234 | -215 | lipoprotein-related protein | 0.19 |
| VPBB_0953 | VP1002 | 4.29 | -105 | -86 | hypothetical protein | 7.84 |
| VPBB_1242 | VP1318 | 3.28 | -73 | -54 | hypothetical protein | 0.08 |
| VPBB_1302 | VP1385 | 4.68 | -269 | -250 | cell wall endopeptidase, family M23 M37 | 5.31 |
| VPBB_1309 | VP1393 | 5 | -211 | -192 | typeVI secretion Hcp protein, biofilm development | 26.72 |
| VPBB_1315 | VP1400 | 6.94 | -59 | -40 | hypothetical protein | 17.45 |
| VPBB_1325 | VP1410 | 6.35 | -330 | -311 | hypothetical protein | 10.89 |
| VPBB_1336 | VP1422 | 3.63 | -54 | -35 | SM-20-related protein | 6.51 |
| VPBB_1422 | VP1517 | 4 | -293 | -274 | hypothetical protein | 0.03 |
| VPBB_1494 | VP1634 | 6.12 | -330 | -311 | agglutination protein | 0.12 |
| VPBB_1560 | VP1701 | 6.7 | -256 | -237 | type III secretion regulator ExsC | 12.7 |
| VPBB_1730 | VP1879 | 8.05 | -119 | -100 | serine transporter | 5.15 |
| VPBB_1732 | VP1881 | 7.35 | -43 | -24 | EAL family protein | 5.97 |
| VPBB_1745 | VP1904 | 4.37 | -88 | -69 | methyl-accepting chemotaxis protein | 9.12 |
| VPBB_1805 | VP1966 | 4.08 | -28 | -9 | proton glutamate symport protein | 6.37 |
| VPBB_1851 | VP2015 | 8.84 | -84 | -65 | cytochrome c4 | 128.99 |
| VPBB_1859 | VP2023 | 6.24 | -200 | -181 | dTDP-glucose 4,6-dehydratase | 0.19 |
| VPBB_1979 | VP2159 | 11.52 | -102 | -83 | methyl-accepting chemotaxis protein | 6.01 |
| VPBB_2053 | VP2235 | 6.13 | -190 | -171 | polar flagellar biosynthesis protein FlhA | 6.77 |
| VPBB_2144 | VP2329 | 4.62 | -85 | -66 | multidrug efflux pump component MtrF | 13.13 |
| VPBB_2451 | VP2631 | 8.72 | -71 | -52 | HD-GYP family protein | 8.87 |
| VPBB_2677 | VP2827 | 4.17 | -35 | -16 | methyl-accepting chemotaxis protein | 5.91 |
| VPBB_2845 | VP3014 | 3.65 | -253 | -234 | putative signal peptide protein | 14.3 |
| VPBB_A0232 | VPA0253 | 7.98 | -85 | -66 | putative transport protein | 6.84 |
| VPBB_A0412 | VPA0457 | 7.36 | -165 | -146 | hypothetical protein | 0.18 |
| VPBB_A0414 | VPA0458 | 9.34 | -373 | -354 | hypothetical protein | 0.19 |
| VPBB_A0415 | VPA0459 | 9.34 | -179 | -160 | surface-induced secreted collagenase | 5.02 |
| VPBB_A0446 | VPA0491 | 9.14 | -162 | -143 | methyl-accepting chemotaxis protein | 5.53 |
| VPBB_A0464 | VPA0511 | 6.23 | -125 | -106 | methyl-accepting chemotaxis protein | 23.68 |
| VPBB_A0470 | VPA0518 | 3.09 | -237 | -218 | GGDEF & EAL family protein | 0.12 |
| VPBB_A0515 | VPA0568 | 5.3 | -59 | -40 | hypothetical protein | 0.12 |
| VPBB_A0559 | VPA0612 | 6.49 | -133 | -114 | methyl-accepting chemotaxis protein | 11.3 |
| VPBB_A0688 | VPA0747 | 8.52 | -92 | -73 | MSHA pilin protein MshA | 16.87 |
| VPBB_A0910 | VPA1000 | 4.53 | -82 | -63 | methyl-accepting chemotaxis protein I | 5 |
| VPBB_A0933 | VPA1027 | 9.84 | -57 | -38 | putative cytoplasmic protein USSDB7A | 0.01 |
| VPBB_A0949 | VPA1043 | 9.17 | -89 | -70 | hypothetical protein | 0.02 |
| VPBB_A0950 | VPA1044 | 9.17 | -250 | -231 | protein kinase | 0.02 |
| VPBB_A0985 | VPA1081 | 6.16 | -68 | -49 | hypothetical protein | 17.09 |
| VPBB_A0994 | VPA1091 | 6.39 | -259 | -240 | hypothetical protein | 5.93 |
| VPBB_A1103 | VPA1203 | 3.05 | -37 | -18 | hypothetical protein | 0.1 |
| VPBB_A1105 | VPA1205 | 6.64 | -191 | -172 | acetoacetyl-CoA reductase | 0.01 |
| VPBB_A1334 | VPA1461 | 10.48 | -308 | -289 | phosphate ABC transporter periplasmic phosphate-binding protein PstS | 8.4 |
| VPBB_A1335 | VPA1462 | 10.48 | -43 | -24 | methyl-accepting chemotaxis protein I (chemoreceptor protein) | 10.9 |
| VPBB_A1362 | VPA1492 | 3.6 | -210 | -191 | methyl-accepting chemotaxis protein I (serine chemoreceptor protein) | 6.41 |
| VPBB_A1381 | VPA1513 | 10.86 | -260 | -241 | aminotransferase ScrA | 11.15 |
| VPBB_A1407 | VPA1540 | 4.89 | -192 | -173 | FliM lateral flagellar motor protein | 6.4 |
| VPBB_A1415 | VPA1548 | 8.77 | -101 | -82 | LafA lateral flagellin protein | 153.3 |
| VPBB_A1416 | VPA1550 | 7.76 | -266 | -247 | FliD lateral flagellar distal cap protein | 321.76 |
| VPBB_A1434 | VPA1570 | 9.94 | -81 | -62 | hypothetical protein | 11.37 |
| VPBB_A1461 | VPA1598 | 3.38 | -301 | -282 | surface-induced N-acetyl glucosamine-Chitin binding protein GpbA | 20.32 |
| VPBB_A1499 | VPA1635 | 7.58 | -281 | -262 | ornithine decarboxylase | 18.23 |
| VPBB_A1512 | VPA1649 | 6.51 | -303 | -284 | surface-induced metalloendoprotease LytM (M23 family) | 82.29 |
| VPBB_A0413^d^ |  | 7.8 | -412 | -393 | hypothetical protein | 0.11 |

**^a^** Paster scores of three or higher required

**^b^** Regulated five-fold or more by OpaR

^c^ RNA-Seq data is fold change of the *opaR^+^* strain gene expression divided by the ∆*opaR1* strain gene expression. Error for the ratios of normalized gene expression levels were conservatively estimated using the standard deviation ratios across the majority of genes with less than 4-fold change. Standard deviation for chromosome 1 genes is ±1.54 and for chromosome 2 gene it is ±1.59.

^d^ Unique to BB22OP
